# Isolation and In Vitro Activity of Sesquiterpene Lactones from *Eremanthus crotonoides* as SARS-CoV-2 Protease Inhibitors and Cytotoxic Agents

**DOI:** 10.3390/molecules30204053

**Published:** 2025-10-11

**Authors:** Patricia Homobono Brito de Moura, Natalie Giovanna da Rocha Ximenes, Beatriz Bastos Santos, Carla Monteiro Leal, Larissa Esteves Carvalho Constant, Stephany da Silva Costa, Shaft Corrêa Pinto, Michelle Frazao Muzitano, Diego Allonso, Ludger A. Wessjohann, Ivana Correa Ramos Leal

**Affiliations:** 1 Laboratory of Natural products and Biological Assays, Natural Products and Food Department, Pharmacy Faculty, Center of Health Sciences, Federal University of Rio de Janeiro (UFRJ), Rio de Janeiro 21941-902, RJ, Brazil; patricia.homobono@gmail.com (P.H.B.d.M.); natalieximenes@gmail.com (N.G.d.R.X.); beatrizbastos139@gmail.com (B.B.S.); carlam.leal@yahoo.com.br (C.M.L.); 2 Leibniz Institute of Plant Biochemistry, Department of Bioorganic Chemistry, Weinberg 3, 06120 Halle, Germany; 3 Laboratory of Biotechnology and Tissue Bioengineering, Institute of Biophysics Carlos Chagas Filho, Center of Health Sciences, Federal University of Rio de Janeiro (UFRJ), Rio de Janeiro 21941-902, RJ, Brazil; larissaconstant@biof.ufrj.br (L.E.C.C.); stephanycosta@biof.ufrj.br (S.d.S.C.); 4 Laboratory of Bioatives Products, Institute of Pharmaceutical Sciences, Federal University of Rio de Janeiro (UFRJ), Macaé 27933-378, RJ, Brazil; shaftcp@yahoo.com.br (S.C.P.); mfmuzitano@gmail.com (M.F.M.); 5 Department of Pharmaceutical Biotechnology, Pharmacy Faculty, Center of Health Sciences, Federal University of Rio de Janeiro (UFRJ), Rio de Janeiro 21941-902, RJ, Brazil; diegoallonso@gmail.com

**Keywords:** centratherin, cytotoxicity, *Eremanthus crotonoides*, goyazensolide, sesquiterpene lactones, SARS-CoV-2

## Abstract

The Jurubatiba Sandbank National Park (PARNA Jurubatiba) is an ecological reserve characterized by harsh environmental conditions, including low rainfall, high sun exposure, and sandy soil. Among its native vegetation, *Eremanthus crotonoides* stands out for its richness in flavonoids, phenolic compounds, and sesquiterpene lactones. The objective of this study was to isolate and quantify sesquiterpene lactones from this species using ^1^H NMR and to investigate their anti-SARS-CoV-2 potential and cytotoxicity against cancer cells. UPLC-(ESI)-MS/MS analyses enabled metabolite annotation, and semi-preparative HPLC-DAD allowed the isolation of centratherin and goyazensolide, which were identified by 1D and 2D NMR. In vitro assays showed that centratherin at 10 µM concentration reduced the viability of PC-3 and HCT-116 cancer cells by 100%, while goyazensolide had no noteworthy effects. Furthermore, enzymatic inhibition assays on SARS-CoV2 targets revealed that centratherin exhibited a lower apparent IC_50_ of 12 µM against PL^pro^, while goyazensolide was more active against 3CL^pro^, with an IC_50_ of 71 µM. Notably, the dichloromethane fraction demonstrated promising activity against both enzymes, with IC_50_ values of 30 µM for PL^pro^ and 11 µM for 3CL^pro^. This study reports, for the first time, the isolation of goyazensolide from *E. crotonoides* and highlights the potential of both sesquiterpene lactones as SARS-CoV-2 enzyme inhibitors. In contrast to centratherin, goyazensolide fortunately had almost no cytotoxic effects at inhibition concentration on the cells tested. This shows that anticancer and anti-SARS effects can be separated and should have different SARs, an important prerequisite for further development.

## 1. Introduction

**Figure 1:**
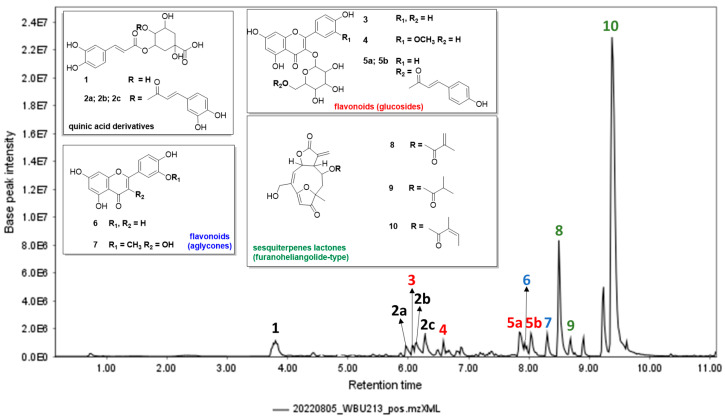
UPLC-(ESI)-MS/MS total ion chromatogram of the crude extract of *E. crotonoides* from 0 to 11 min, highlighting the annotated compounds: chlorogenic acid (**1**), caffeoylquinic acid isomers (**2a**–**2c**), quercetin-3-*O*-glucoside (**3**), isorhamnetin-3-*O*-glucoside (**4**), tiliroside (**5**), luteolin (**6**), isorhamnetin (**7**), goyazensolide (**8**), budlein A isobutyrate (**9**), and centratherin (**10**). Note: Axis labels are displayed in MZmine format (e.g., 2.0E6 corresponds to 2.0 × 10^6^).

*Eremanthus crotonoides* (DC.) Sch.Bip. (Asteraceae) is a native Brazilian species that can be found in various regions of the northeastern and southeastern parts of the country [1]. Several studies have investigated the biological properties of this species. For example, the ethyl acetate fraction obtained from its leaves has shown potential as an antidiabetic agent, with flavonoids and caffeoylquinic acid derivatives identified as α-glucosidase inhibitors [2]. Previous research has also examined the ethanolic extract and dichloromethane fraction from the leaves, as well as on the isolated compound centratherin [3]. All of these samples exhibited cytotoxic activity against glioblastoma multiforme, with centratherin (**10**, Figure 1), a sesquiterpene lactone, showing IC_50_ values comparable to the commercial anticancer drug doxorubicin [3].

Additionally, our research group has conducted two studies to date. The first was a bio-guided investigation that identified the dichloromethane fraction as bactericidal against methicillin-resistant *Staphylococcus aureus* (MRSA, ATCC 33591) at a concentration of 128 μg/mL. In this study, centratherin inhibited a clinical isolate of *S. aureus* at 32 µg/mL and demonstrated antibiofilm activity at 128 µg/mL [4]. The second study focused on the dichloromethane fraction, which showed MIC_50_ values of 0.95 ± 1.08 μg/mL against *Mycobacterium bovis* BCG and 2.17 ± 1.11 μg/mL against *M. tuberculosis* H37Rv [5]. Furthermore, this fraction inhibited NO and TNF-α production in LPS-stimulated macrophages, while remaining non-cytotoxic.

All these findings regarding the intriguing chemical and biological profile of *E. crotonoides* motivated this study, which aimed to investigate the main constituents of this species, specifically the sesquiterpene lactones. To this end, the chemical profile of the crude extract was explored using Ultra-Performance Liquid Chromatography coupled with Electrospray Ionization Tandem Mass Spectrometry (UPLC-(ESI)-MS/MS). Additionally, furanoheliangolide-type sesquiterpene lactones were isolated by semi-preparative High-Performance Liquid Chromatography with Diode Array Detection (HPLC-DAD), identified by one-dimensional (1D) and two-dimensional (2D) Nuclear Magnetic Resonance (NMR), and quantified by quantitative proton NMR (*q*^1^H NMR) from the dichloromethane fraction (FDCM). The cytotoxicity of the *E. crotonoides* crude extract (EBEC), dichloromethane fraction, centratherin (**10**, Figure 1), and goyazensolide (**8**, Figure 1) against human carcinoma cells was evaluated, along with their potential enzymatic inhibition against recombinant papain-like protease (rPL^pro^) and 3-chymotrypsin-like protease (3CL^pro^).

## 2. Results and Discussion

Research on species from Brazil’s coastal restinga ecosystem is particularly important due to the distinctive adaptations of its flora, which often result in the production of specialized secondary metabolites with potential pharmacological applications. The analysis of *E. crotonoides*, a species native to this unique habitat and notable for its diverse chemical profile, enabled the identification of several compounds with promising bioactive properties. These findings encompass extraction, detailed chemical characterization, and bioactivity assays, highlighting the pharmacological relevance of these metabolites.

### 2.1. Extraction Yield and Fractionation of Leaves

From 34 g of dried leaves, 2.7 g of freeze-dried crude extract was obtained after extraction with ethanol. Of this total, 1 g was subjected to liquid–liquid partitioning, yielding fractions in *n*-hexane, dichloromethane, and ethyl acetate with yields of 7.4%, 15.2%, and 4.8%, respectively, relative to the dried extract weight. For this study, only the dichloromethane fraction and the crude extract were selected, as they showed greater potential for subsequent bioactivity assays.

### 2.2. Chemical Characterization of E. crotonoides Crude Extract

UPLC-(ESI)-MS/MS analysis of the crude extract of *E. crotonoides* (EBEC) enabled the characterization of 10 compounds (Figure 1 and Table S1). Overall, the extract appears to be rich in quinic acid derivatives, flavonoids, and furanoheliangolide-type sesquiterpene lactones. Based on dereplication studies using the GNPS platform, the literature, and fragmentation pathway analysis, the annotated compounds were chlorogenic acid (**1**), caffeoylquinic acid (**2**), quercetin-3-*O*-glucoside (**3**), isorhamnetin-3-*O*-glucoside (**4**), tiliroside (**5**), luteolin (**6**), isorhamnetin (**7**), goyazensolide (**8**), budlein A isobutyrate (**9**), and centratherin (**10**). Additionally, all the phenolic compounds mentioned were previously identified and isolated in the ethyl acetate fraction of *E. crotonoides* [2].

The MS^2^ spectrum of compound **1** (*m*/*z* 355.1019 [M+H]^+^) showed a fragment ion at *m*/*z* 163.0376 [caffeic acid–H_2_O+H]^+^, indicating a neutral loss of 192 Da, characteristic of the quinic acid moiety, leading to the annotation as chlorogenic acid (Table S1; Figure S1) [6]. For the isomeric forms of compound **2** (**2a**: *m*/*z* 517.1345 [M+H]^+^; **2b**: *m*/*z* 517.1326 [M+H]^+^; **2c**: *m*/*z* 517.1337 [M+H]^+^, a fragment ion at *m*/*z* 163.0378 was also observed, corresponding to the losses of 162 (caffeic acid–H_2_O) and 192 Da (quinic acid moiety). Thus, compound **2** was annotated as caffeoylquinic acid (Table S1; Figures S2, S3 and S5) [7].

Compounds **3** (*m*/*z* 465.1027 [M+H]^+^) and **4** (*m*/*z* 479.1178 [M+H]^+^) exhibited fragment ions in their MS^2^ spectra corresponding to a neutral loss of 162 Da, suggesting the presence of a hexose moiety. These were identified as the *O*-glycosylated flavonoids quercetin-3-*O*-glucoside and isorhamnetin-3-*O*-glucoside, respectively (Table S1 and Figures S4–S6) [8,9]. Furthermore, compound **5** (*m*/*z* 595.1450 [M+H]^+^) showed in fragment ions at *m*/*z* 309.0968 and *m*/*z* 287.0551, corresponding to coumaroyl glucoside moiety and kaempferol aglycone, respectively, and was annotated as tiliroside (Table S1 and Figure S7) [10].

The spectrum of compound **6** (*m*/*z* 287.0565 [M+H]^+^) showed characteristic fragments ions at *m*/*z* 241.0481, 213.0520, and 153.0166, typical for luteolin. The fragment at *m*/*z* 153.0166 corresponds to the ^1,3^A^+^ Retro-Diels-Alder (RDA) fragment of this flavonoid (Table S1 and Figure S8) [11]. For compound **7** (*m*/*z* 317.0670 [M+H]^+^) the MS/MS spectrum displayed a fragment ion at *m*/*z* 302.0404, indicating a loss of 15 Da, characteristic of a methyl group, suggesting that this compound corresponds to isorhamnetin (Table S1 and Figure S9) [12].

**Scheme 1:**
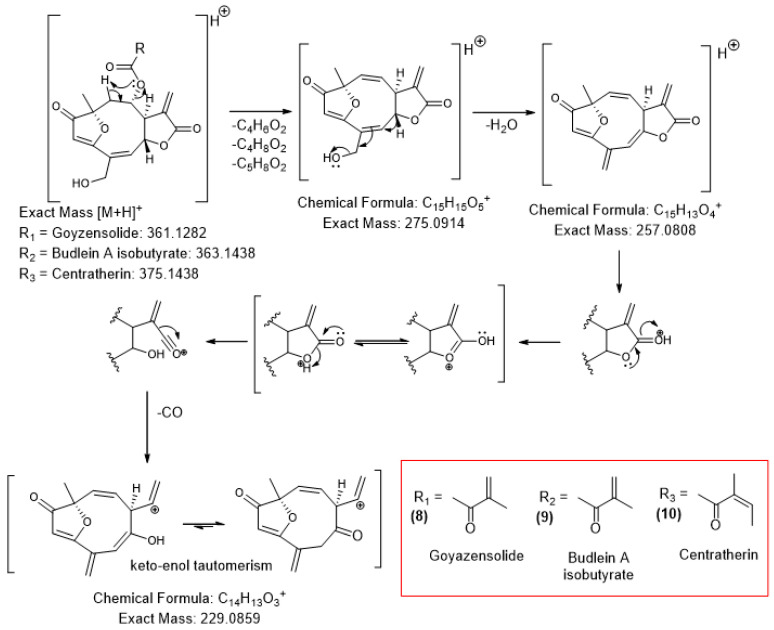
Proposed fragmentation pathway of furanoheliagolide-type sesquiterpene lactones goyazensolide (**8**), budlein A isobutyrate (**9**), and centratherin (**10**). The substituents displayed in the red box correspond to the R (R_1_, R_2_, R_3_) groups indicated in the chemical structures.

The three characterized sesquiterpene lactones exhibit similar fragmentation patterns, with their MS^2^ spectra presented in Figures S10–S12. The proposed fragmentation pathway, depicted in Scheme 1, suggests that all three compounds share the same main skeleton, with the daughter ion *m*/*z* 275.09 originating from the loss of the radical attached to C-8. This allows the characterization of compound **8** as goyazensolide, compound **9** as budlein A isobutyrate, and compound **10** as centratherin. Further successive fragmentations include the loss of water (*m*/*z* 257.08) and ultimately carbon monoxide (CO) (*m*/*z* 229.08) from the lactonic ring, based on fragmentations studies of five-membered lactones by electrospray ionization [13].

This represents the first proposed fragmentation pathway for the sesquiterpene lactones under study, as well as the first characterization of budlein A isobutyrate in *E. crotonoides*, which had previously been described only in species of *Helianthus* [14,15,16], also from the Asteraceae family.

In summary, concerning the characterization of the crude extract of the studied species, GNPS facilitated the dereplication of phenolic acids and flavonoids, supported by high cosine values (0.93–0.99, Table S1). For sesquiterpene lactones, however, the results were less straightforward: goyazensolide showed only a moderate score (cosine = 0.73, Table S1) while budlein A isobutyrate and centratherin showed no library matches. This study therefore illustrates that, although GNPS is a useful tool to accelerate dereplication, its application must be combined with manual inspection of MS^2^ data to ensure reliable identification, especially for compounds underrepresented in spectral libraries. Importantly, this represents the first dereplication study reported for *Eremanthus crotonoides*, providing a chemical baseline that supported the subsequent biological assays.

### 2.3. Isolation and Identification of Sesquiterpene Lactones

Following the chemical characterization of the species, efforts were focused on the analysis of the dichloromethane extract of *E. crotonoides* due to the promising nature of sesquiterpene lactones as bioactives. Figure S13 shows the overlapped ^1^H NMR spectra of the crude extract and the dichloromethane fraction, highlighting the main peaks in each sample.

The dichloromethane fraction of *E. crotonoides* (FDEC) was subjected to isolation by semi-preparative HPLC-DAD monitored at λ = 269 nm, yielding six collected peaks as shown in Figure S14. All these peaks were analyzed by electrospray ionization mass spectrometry (direct injection) in both positive and negative ionization modes. Peaks 1 (rt = 4.02 min) and 2 (rt = 4.48 min) ionized more effectively in negative mode, showing [M−H]^−^ ions at *m*/*z* 285.3 and *m*/*z* 315.2, respectively, and exhibited very similar ultraviolet profiles. The *m*/*z* 285.3 ion is suggestive of kaempferol, which had already been identified during dereplication studies. Peaks 3 to 6 showed better ionization in positive mode, with *m*/*z* values of 383.1 (peak 3, rt = 4.98 min, [M+Na]^+^), 385.2 (peak 4, rt = 5.15 min, [M+Na]^+^), 397.7 (peak 5, rt 6.12 min, [M+Na]^+^), and 397.5 (peak 6, rt = 6.38 min, [M+Na]^+^), and also displayed similar ultraviolet profiles.

All peaks were inspected by ^1^H NMR, and peaks 3 and 6 showed the best spectral resolution; these were therefore selected for comprehensive 1D and 2D NMR analysis (Figures S15–S27). Subsequent isolation afforded goyazensolide (11 mg) and centratherin (38 mg), whose structures were assigned from ^1^H and ^13^C NMR data (Table 1). GNPS library matching had previously suggested the presence of goyazensolide in *E. crotonoides*; the present work documents its first isolation and full NMR characterization from this species. Centratherin had been reported in this species by our group [4]. The 1D and 2D NMR datasets are consistent with literature values for these sesquiterpene lactones isolated from other Asteraceae species, including Soares et al. (2012) [17] and Santos Junior et al. (2015) [18], the latter establishing the absolute configuration of centratherin by ECD and supporting the assignments presented here.

As previously shown in Figure S14, the HPLC-DAD analysis monitored at λ = 269 nm identified centratherin (**10**) and goyazensolide (**8**) as main constituents of FDEC. Both compounds were quantified by ^1^H NMR (10 mg/mL). This non-destructive technique offers several advantages, such as shorter analysis time compared to conventional chromatographic methods, albeit with lower sensitivity. Additionally, unlike chromatographic or MS-based methods, the detection of compounds by ^1^H NMR does not dependent on their polarity, ionization efficiency, or light absorption. 

**Figure 2:**
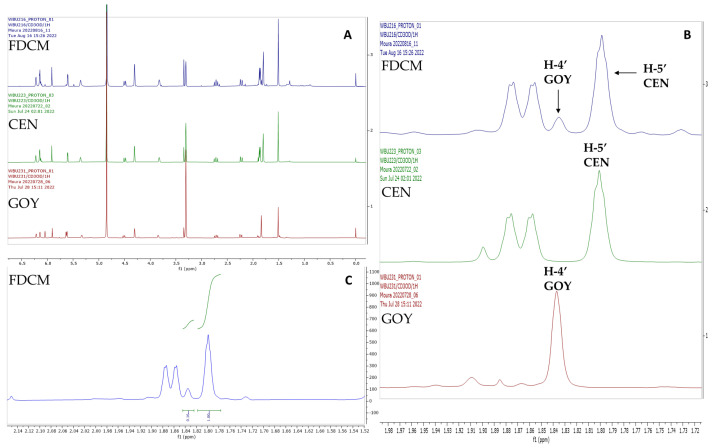
Quantitative ^1^H NMR. Overlap of spectra (δ_H_ 0.0 to 6.75 ppm) of the dichloromethane fraction (FDCM), goyazensolide (GOY), and centratherin (CEN) (**A**). Expansion (δ_H_ 1.72 to 1.98 ppm) of compounds and fraction spectra to highlight H-4′ of goyazensolide and H-5′ of centratherin in the dichloromethane fraction, isolated goyazensolide and centratherin (**B**) and expansion (δ_H_ 1.52 to 2.14 ppm) of ^1^H NMR spectrum of dichloromethane fraction (**C**).

Figure 2 presents the ^1^H NMR spectra of centratherin, goyazensolide and FDEC. The non-overlapping signals of H-4′ of goyazensolide and H-5′ of centratherin allow quantification throughout signal integration. In this case, the integrated signals show a ratio of 0.16:1 (goyazensolide–centratherin), indicating that centratherin is present at approximately 6.25 times the concentration of goyazensolide.

### 2.4. Cytotoxicity Against Human Carcinoma Cells

The cell viability of carcinoma cells tested in vitro with EBEC, FDEC, centratherin and goyazensolide was evaluated using MTT and crystal violet (CV) assays, and the results are presented in Table 2. Both EBEC and FDEC, at a concentration of 50 µg/mL, showed promising activity by reducing cancer viability by 100%. Regarding the cytotoxicity of the isolated compounds, goyazensolide was unable to decrease cell viability after 48 h at concentrations ≤ 10 µM; however, centratherin was able to reduce cell viability of both PC-3 and HCT-116 cell lines by 100%. Therefore, it can be inferred that the cytotoxic activity of EBEC and FDEC against the evaluated strains is primarily correlated with centratherin.

The α-methylene-γ-lactone ring of sesquiterpene lactones (SQLs) is well recognized for its pivotal role in imparting cytotoxic effects against (cancer) cells. For instance, centratherin and goyazensolide have previously demonstrated promising activity against glioma (U251 and U87-MG) [2], lung carcinoma (NCI-H187) [19], and colon cancer (HT-29) [20] cell lines, with results comparable to commercial standards. However, to the best of our knowledge, this is the first study to evaluate the anticancer potential of *E. crotonoides* and its sesquiterpene lactones against PC-3 and HCT-116 cells. This novel insight enriches the scientific literature and further highlights *E. crotonoides* as a potential source of anticancer agents, and with some cell type specificity of the different derivatives.

### 2.5. Enzymatic Inhibition Against PL^pro^ and 3CL^pro^

PL^pro^ is one of the SARS-CoV-2 proteases responsible for processing the viral polyprotein (pp1ab) into single, functionally active non-structural proteins. This protease is a domain located within the nsp3 protein and exhibits papain-like activity, performing specific cleavages between nsp1 and nsp2, nsp2 and nsp3, and between nsp3 and nsp4 in pp1ab. Inhibition of PL^pro^ activity results in deficient polyprotein processing, consequently blocking virus replication. On the other hand, the 3-Chymotrypsin-like protein (3CL^pro^) plays a fundamental role in peptide chain processing by cleaving the C-terminal domain at 11 sites, producing enzymes and structural proteins essential for viral function [21]. Given the significance of these proteases for the SARS-CoV-2 life cycle, they represent targets for the development of antiviral compounds [22].

**Figure 3:**
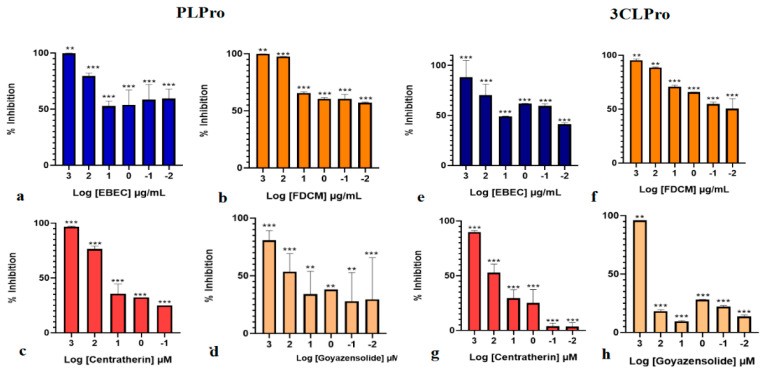
Effect of (**a**) the crude extract (EBEC) and (**b**) dichloromethane fraction (FDCM) from the leaves of *Eremanthus crotonoides*, (**c**) centratherin and (**d**) goyazensolide on the PL^pro^ enzyme; and (**e**) the crude extract (EBEC), (**f**) dichloromethane fraction (FDCM), (**g**) centratherin, and (**h**) goyazensolide on the 3CL^pro^ enzyme. Enzymatic inhibition percentages were determined using the FRET assay. The bars are arranged in descending according to the logarithms of tested concentrations (1000, 100, 10, 1, 0.1, 0.01, 0.001, and 0.0001 µg/mL for the extract and fraction; 1000, 100, 10, 1, 0.1, and 0.01 µM for centratherin and goyazensolide). Values are presented as mean ± standard deviation (n = 2). Statistical significance is indicated as ** *p* < 0.05 and *** *p* < 0.0001. The control consisted of substrate, buffer, and the corresponding enzyme only.

The inhibitory effects of EBEC and FDCM against both proteases are shown in Table S3, Figure 3a,b,e,f. The samples exhibited strong inhibitory potential, with a clear dose-dependent response. At the highest concentrations tested (1 µg/mL), the inhibition percentage reached nearly 100% against PL^pro^ and 88–95% against 3CL^pro^. Interestingly, the dichloromethane fraction demonstrated particularly promising activity against both enzymes, with IC_50_ values of 30 and 10.7 µg/mL, respectively. This behavior can be attributed to the chemical profile of this fraction, which is primarily composed of sesquiterpene lactones (SQLs). This class of compounds is notable for its diverse biological properties, including antiviral activity [23,24,25].

The biological activities of SQLs are often attributed to the presence of the α-methylene-γ-butyrolactone moiety, which reacts with nucleophiles through a Michael addition and acts as an alkylating agent. Therefore, these compounds are believed to act via covalent bonding to cysteine residues in target proteins, as well as through protein inhibition and enzymatic conjugation with glutathione [20]. The latter process, though, can also lead to inactivation. To evaluate the potential of *E. crotonoides* STLs, centratherin and goyazensolide were isolated and tested for their PL^pro^ and 3CL^pro^ inhibitory activity.

As shown in Figure 3c,d,g,h and Table S3, both compounds have inhibitory potential against the proteases, with a clear dose-dependent response. Interestingly, the two SQLs displayed distinct behaviors against the analyzed proteases. Centratherin demonstrated superior activity against PL^pro^, achieving 97% inhibition at the highest concentration tested (IC_50_ 11.9 µM), whereas goyazensolide was more effective against 3CL^pro^, showing 96% inhibition at the highest concentration (IC_50_ 71 µM). These values are consistent with those reported for other natural products with significant inhibitory potential against PL^pro^ [26,27]. To the best of our knowledge, there are no previous reports on the antiviral activity of centratherin, while goyazensolide has been described as an important covalent inhibitor of importin-5, a key transporter involved in viral replication [19].

It is important to emphasize that the results of this study demonstrate that both compounds possess the ability to inhibit the two proteases under investigation, indicating promising antiviral potential by targeting two distinct pathways: the specific cleavages in the polyprotein (pplab) and the cleavage of the C-terminal domain. This dual inhibitory action may help explain the strong results obtained for the dichloromethane fraction of *E. crotonoides* leaves, which is rich in sesquiterpene lactones such as centratherin and goyazensolide. Future molecular docking analyses could provide complementary insights into the binding interactions of centratherin and goyazensolide with PL^pro^ and 3CL^pro^, helping to rationalize the inhibitory profiles observed in this study.

## 3. Materials and Methods

### 3.1. Collection, Preparation and Extraction of E. crotonoides

The species was identified in situ by the botanist and plant taxonomy specialist Tatiana Ungaretti Paleo Konno, and a voucher specimen (RFA-38749) was deposited in the herbarium of the Instituto de Biodiversidade e Sustentabilidade (NUPEM) at the Federal University of Rio de Janeiro, Macaé, Brazil. The collection was authorized under SISBIO/ICMBio No 62.455-11, and the research was approved by SISGEN/MMA No: AAA989F. Aerial parts of the *E. crotonoides* were harvested in the National Park of Jurubatiba Sandbank (PARNA Jurubatiba, coordinates: 22.19970° S 41.47112° W, Quissamã, Rio de Janeiro, Brazil) in May 2022. Healthy leaves were selected and dried in a circulation air oven at 40 °C for three days. After drying, the leaves were powdered using a domestic processor and extracted with ethanol (99.5%). The extraction was performed over five days, with the solvent (300 mL) renewed every 24 h. Following extraction, the solution was concentrated using a rotary evaporator, and the residual extract was freeze-dried for five days.

### 3.2. Fractionation of Crude Extracts Using the Liquid–Liquid Partitioning

The crude extract from the leaves of *E. crotonoides* (EBEC) was fractionated using solvents of increasing polarity: *n*-hexane, dichloromethane, and ethyl acetate, yielding three fractions. Among these, only the dichloromethane fraction of *E. crotonoides* (FDEC) was used in this present work due to its promising chemical profile.

### 3.3. Metabolic Profiling of the Crude Extract by High-Resolution UPLC-MS^n^

The crude extract (5 mg/mL) was diluted in methanol–water (4:1), centrifuged (2000 rpm), and the supernatant was analyzed by high-resolution UHPLC-MS^n^. Chromatographic separation was performed using an ACQUITY UPLC I-Class UHPLC System (Waters GmbH, Eschborn, Germany) under the following gradient conditions: 5%B-2 min; 5%B to 95%B-2 to 18 min; 95%B to 5%B-18 to 23 min, with a flow rate of 0.4 mL/min. The column used was an EC 150/2 Nucleoshell (C18-phase, I.D. 2 mm, 150 mm, 2.7 µm, Macherey Nagel, Düren, Germany) maintained at 40 °C. Eluents A and B were 0.3 mmol/L aqueous ammonium formate and acetonitrile, respectively. The separation system was coupled to a high-resolution TripleTOF 6600-1 mass spectrometer (AB Sciex™, Framingham, MA, USA) equipped with a DuoSpray ESI/APCI ion source, operating in positive and negative modes.

Precursor ions were selected in Data Dependent Acquisition (DDA) mode. MS experiments were conducted in TOF-scan mode with an accumulation time of 100 ms covering a *m*/*z* range of 65–1250. Tandem mass spectrometric (MS^2^) experiments were performed in SWATH mode, with the overall range (*m*/*z* 65–1250) divided into 48 windows of 26 *m*/*z* each, with a 1 *m*/*z* overlap. Each *m*/*z* window was acquired with a 20 ms accumulation time at a collision energy (CE) of −35 V, using a collision energy spread (CES) of 15 V and a declustering potential (DP) of −35 V. Nitrogen was used as the collision-activated dissociation (CAD) gas.

Raw data (*.wiff format) were converted to *.mzML using MSConvert (ProteoWizard; https://proteowizard.sourceforge.io/, accessed on 5 September 2022) and processed in MZmine version 2.53 (Table S2). Dereplication was performed using the GNPS platform (Global Natural Products Social Molecular Networking; https://gnps.ucsd.edu/ProteoSAFe/status.jsp?task=4f12c4f9a8f640dcb32c1633ded31735, accessed on 10 September 2022) in combination with literature data and manual interpretation of MS^2^ fragmentation patterns.

### 3.4. Isolation of Compounds

The isolation step was performed using a semi-preparative approach on an Agilent 1260 HPLC-DAD system, with the column maintained at 40 °C and monitored at wavelengths λ = 210 and 269 nm. Separations were conducted on a reverse phase C8 column (VarioPrep VP125/10 nucleodur 100_5 C8 ec, 125 × 10 MM). The injection volume was 40 µL (50 mg/mL, in methanol) with a flow rate of 7 mL/min. The mobile phase consisted of water (A) and acetonitrile (B), with the following gradient: 30%B–70%B–from 0 to 10 min; 70%B–100%B–from 10 to 12 min; 100%B–30%B–from 12 to 15 min.

### 3.5. NMR Analysis

One-dimensional (1D) and two-dimensional (2D) ^1^H and ^13^C spectra were acquired using an Agilent^®^ DD2 400 MHz (9.4 Tesla) spectrometer (Santa Clara, CA, USA) operating at 400 MHz (1H) and 100 MHz (13C), or an Agilent VNMRS 600 (Varian, Palo Alto, CA, USA) operating at 599.83 MHz (1H) and 149.84 MHz (13C). The VNMRS 600 was used exclusively for quantitative analysis.

The 1D (^1^H, ^13^C) and 2D (^1^H-^13^C) gHSQCAD; (^1^H-^13^C) gHMBCAD; (^1^H-^13^C) gH2BCAD; (^1^H-^13^C) DQFCOSY; (^1^H-^1^H) zTOCSY and (^1^H-^1^H) ROESYAD NMR spectra were acquired using standard CHEMPACK 8.1 pulse sequences implemented in the VNMRJ 4.2A spectrometer software. All spectra were recorded in CD_3_OD at +25 °C, and quantitative CD_3_OD was used for quantitative measurements. ^1^H and ^13^C chemical shifts were referenced to the internal standard hexamethyl disiloxane (δ_H_ 0.062 ppm; δ_C_ 1.96 ppm). The following parameters were applied: TOCSY mixing time = 80 ms; ROESY mixing time = 300 ms; HSQC optimized for ^1^*J*_CH_ = 146 Hz; HMBC optimized for ^n^*J*_CH_ = 8 Hz.

### 3.6. Biological Activities

#### 3.6.1. Cytotoxic Activity Against Cancer Cells

The cytotoxicity of crude extract and isolated compounds were evaluated against the human tumor cell lines PC-3 (prostatic carcinoma cell) and HCT-116 (colorectal carcinoma cell). Cell maintenance was performed as described in the literature [28]. The extract and isolated compounds were tested at the concentrations of 0.05 and 50 μg/mL and 0.01 and 10 μM, respectively. Saponin digitonin (100 µM), a very potent permeabilizer of cell membranes, was used as positive control to induce 0% cell viability after 48 h of treatment.

Cell viability was determined by MTT and crystal violet (CV) assays after 48 h of incubation [29]. Absorbance was measured using an automated microplate reader at 540 nm with a reference wavelength of 670 nm. Results are expressed as percentages relative to untreated control cultures.

#### 3.6.2. Screening of Plant Extract, Fraction and Isolated Compounds for Inhibition of SARS-CoV-2 PL^pro^ and 3CL^pro^ Enzymatic Assay

Recombinant SARS-CoV-2 3CL^pro^ and PL^pro^, expressed in *E. coli* BL21(DE3) pLysS and BL21(DE3) cells (purchased from ThermoFisher, Waltham, MA, USA, USA cat C600003), respectively, were used in a fluorescence resonance energy transfer (FRET) assay. The peptides DABCYL-AVLQ↓SGFRLL-EDANS and DABCYL-ALKG↓GKIV-EDANS were employed as substrates for 3CLpro and PLpro, respectively. The 3CLpro concentration was fixed at 1.5 µM, and the substrate concentration at 50 µM. The crude extract (EBEC) and the dichloromethane fraction (FDCM) from *E. crotonoides* leaves were tested at concentrations ranging from 0.0001 to 1000 µg/mL. The same protocol was applied to the isolated compounds, tested over a range of 0.01 to 1000 µM.

The reaction mixture was incubated in 5 mM NaCl, 20 mM Tris. HCl pH 8.0, 5 mM DTT for 15 min at 37 °C prior to addition of the substrate. For PL^pro^, the enzyme concentration was set in 1 µM, using a reaction buffer containing 150 mM NaCl, 20 mM Tris.HCl pH 8.0, and 5 mM DTT. EDANS emission fluorescence was monitored every 30 s for 45 min, at 37 °C (λexc = 330 nm, λem = 490 nm).

Fluorescence data (RFU) were converted to specific substrate cleavage activity using a fluorescent conversion factor (FEC) previously determined for the EDANS-DABCYL fluorophore pair. Maximum enzyme activity was defined by reactions containing only vehicle (DMSO) and was used to calculate percentage inhibition by the tested samples. The concentration required to inhibit 50% of enzyme activity (IC_50_) was determined, and statistical analysis were performed by one-way ANOVA followed by Tukey’s test, all using GraphPad Prism 8.0.

## 4. Conclusions

In this work, several compounds from *E. crotonoides* collected in the PARNA Jurubatiba restinga were annotated. Among them, furanoheliangolide-type sesquiterpene lactones such as budlein A were characterized, and centratherin and goyazensolide were isolated and identified, with the latter being isolated for the first time in this species in quantities sufficient for unequivocal identification by NMR. Regarding the antitumoral activity of the isolated metabolites, centratherin demonstrated promising cytotoxic potential, completely inhibiting the viability of cancer cells at 10 µM, while goyazensolide had no such effect. Furthermore, enzymatic inhibition assays against SARS-CoV-2 proteases revealed significant activity of both centratherin and goyazensolide against PL^pro^ and 3CL^pro^, along with promising results for the dichloromethane extract, which was shown by quantitative ^1^H NMR to be rich in centratherin. These findings highlight *E. crotonoides* extracts as a promising source of bioactive sesquiterpene lactones with notable anti-SARS-CoV-2 potential. Notably, in goyazensolide, cytotoxic and anti-SARS-CoV-2 activities appear to be structurally unrelated. This work encourages further in-depth exploration and targeted isolation of similar compounds or of semisynthetic derivatives, derived from this species and other members of the *Eremanthus* genus, underscoring the unique pharmacological potential within these plants. Moreover, this study is the first to reveal the anti-SARS-CoV-2 activity of *E. crotonoides* and its sesquiterpene lactones, opening new avenues for phytochemical and biological research in antiviral drug discovery.

## Figures and Tables

**Table 1:** NMR chemical shifts (^1^H and ^13^C) of sesquiterpene lactones isolated from dichloromethane fraction of *E. crotonoides*.

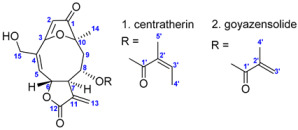				
Position	Centratherin		Goyazensolide	
C	δ ^13^C [ppm]	δ ^1^H [ppm] m (*J* [Hz])	δ ^13^C [ppm]	δ ^1^H [ppm] m (*J* [Hz])
1	207.5	--	207.4	--
2	107.6	5.92 *s*	107.5	5.91 *s*
3	187.1	--	187.0	--
4	137.0	--	137.1	--
5	135.3	6.23 *dt* (3.2; 1.7)	135.2	6.22 *dt* (3.2, 1.7)
6	83.6	5.36 *dq*-like (5.0, 2.4)	83.4	5.33 *dt* (5.0, 2.5)
7	52.3	3.82 *dq*-like (5.5, 2.8)	52.2	3.84 *dq* (5.6, 2.8)
8	74.9	4.49 *dt*-like (11.9, 2.2)	75.2	4.51 *dt* (11.8, 2.3)
9	44.7	9α-2.72 *dd* (13.9, 11.9)	44.5	9α-2.72 *dd* (14.0, 11.8)
		9β-2.23 *dd* (14.0, 1.9)		9β-2.24 *dd* (14.0, 2.0)
10	91.4	--	91.3	--
11	136.0	--	134.9	--
12	171.0	--	170.9	--
13	125.6	5.61 *d* (2.6)	125.7	5.65 *d* (2.7)
		6.15 *d* (3.0)		6.14 *d* (3.1)
14	20.8	1.51 *s* (1.8)	20.8	1.51 *s*, 3H
15	63.2	4.30 *q*-like (2H)	63.2	4.31 *q* (2.2)
1′	168.7	--	168.3	--
2′	128.0	--	137.0	--
3′	141.4	6.15–6.09 *m*	127.1	6.06–6.03 *m*
				5.62 *quint* (1.6)
4′	15.9	1.87 *dq* (7.3, 1.6), 3H	18.1	1.837 *s*, 3H
5′	20.2	1.80 *q* (1.5), 3H	--	--

**Table 2:** Cell viability (%) of prostate cancer PC-3 and colon cancer HCT-116 cells treated for 48 h with EBEC, FDEC, centratherin and goyazensolide, determined by MTT and crystal violet assays. Data represented by the average of the biological triplicates (n = 3) and the standard deviation (±).

Sample	Concentration	PC-3 MTT	PC-3 CV	HCT-116 MTT	HCT-116 CV
EBEC	0.01 µg/mL	126.8 ± 3.0	102.3 ± 2.9	112.0 ± 5.2	96.1 ± 1.3
EBEC	50 µg/mL	0.0 ± 11.1	0.0 ± 2.0	0.0 ± 2.7	0.0 ± 3.0
FDEC	0.05 µg/mL	98.0 ± 2.4	108.6 ± 6.8	105.0 ± 5.5	105.3 ± 6.1
FDEC	50 µg/mL	0.6 ± 31.3	0.0 ± 4.3	0.0 ± 3.0	0.0 ± 6.0
CENT	0.01 µM	100.0 ± 13.5	100.0 ± 3.6	100.0 ± 9.6	114.5 ± 5.6
CENT	10 µM	0.0 ± 11.4	0.0 ± 5.3	0.0 ± 21.7	3.5 ± 9.1
GOYA	0.01 µM	86.6 ± 4.7	89.5 ± 2.8	99.6 ± 5.9	113.0 ± 4.0
GOYA	10 µM	100.0 ± 18.0	91.4 ± 1.7	100.0 ± 5.8	92.1 ± 9.0
DGTN	125 µg/mL (101.7 µM)	0.0 ± 8.6	0.0 ± 7.1	0.0 ± 6.5	0.0 ± 3.3

Abbreviations and units: EBEC, crude extract of leaves from *E. crotonoides*; FDEC, dichloromethane fraction of leaves from *E. crotonoides*; CENT, centratherin; GOYA, goyazensolide; DGTN, digitonin; concentrations for EBEC, FDEC, and DGTN are in µg/mL, and for CENT and GOYA they are in µM.

## Data Availability

The original contributions presented in this study are included in the article and Supplementary Materials. Further inquiries can be directed to the corresponding authors.

## Supplementary Materials

The following supporting information can be downloaded at https://www.mdpi.com/article/10.3390/molecules30204053/s1. Table S1: Compounds characterized by UPLC-(ESI)-MS/MS in the crude extract of *Eremanthus crotonoides* compared to the literature, GNPS (cosine similarity), and considering fragmentation patterns. Table S2: MZmine 2.53 software parameters. Table S3: EBEC, FDCM, centratherin, and goyazensolide from the leaves of the *Eremanthus crotonoides* effects on the PLpro enzyme. Figure S1: Chlorogenic acid MS^2^ spectrum *m*/*z* 355.1019 [M+H]^+^. Figure S2: Caffeoylquinic acid (isomer I) MS^2^ spectrum *m*/*z* 517.1345 [M+H]^+^. Figure S3: Caffeoylquinic acid (isomer II) MS^2^ spectrum *m*/*z* 517.1326 [M+H]^+^. Figure S4: Quercetin-3-*O*-glucoside MS^2^ spectrum *m*/*z* 465.1027 [M+H]^+^. Figure S5: Caffeoylquinic acid (isomer III) MS^2^ spectrum *m/z* 517.1337 [M+H]^+^. Figure S6: Isorhamnetin-3-*O*-glucoside MS^2^ spectrum *m*/*z* 479.1178 [M+H]^+^. Figure S7: Tiliroside MS^2^ spectrum *m*/*z* 595.1450 [M+H]^+^. Figure S8: Luteolin MS^2^ spectrum *m*/*z* 287.0565 [M+H]^+^. Figure S9: Isorhamnetin MS^2^ spectrum *m*/*z* 317.0670 [M+H]^+^. Figure S10: Goyazensolide MS^2^ spectrum *m*/*z* 361.1307 [M+H]^+^. Figure S11: Budlein A isobutyrate MS^2^ spectrum *m*/*z* 363.1448 [M+H]^+^. Figure S12: Centratherin MS^2^ spectrum *m*/*z* 375.1440 [M+H]^+^. Figure S13: Overlapped ^1^H NMR spectra (δ_H_ −0.5 to 8.0 ppm) of the crude extract and the dichloromethane fraction of *E. crotonoides.* Figure S14: HPLC-DAD of dichloromethane fraction monitored at λ = 269 and the UV spectra of their six peaks collected. Figure S15: ^1^H NMR spectra (δ_H_ −0.25 to 6.5 ppm) of goyazensolide. Figure S16: ^13^C NMR spectra (δ_C_ 0.0 to 210.0 ppm) of goyazensolide. Figure S17: HSQC (^1^H-^13^C) NMR spectra (δ_H_ −0.25 to 6.5 ppm; δ_C_ 10 to 145 ppm) of centratherin. Figure S18: HMBC (^1^H-^13^C) NMR spectra (δ_H_ 1.2 to 6.6 ppm; δ_C_ 0.0 to 210 ppm) of centratherin. Figure S19: (^1^H-^1^H) NOESY NMR spectra (δ_H_ 0.0 to 6.5 ppm; δ_H_ 0.0 to 7.0 ppm) of centratherin. Figure S20: (^1^H-^1^H) COSY NMR spectra (δ_H_ 0.0 to 7.0 ppm; δ_H_ 0.0 to 6.5 ppm) of centratherin. Figure S21: (^1^H-^1^H) TOCSY NMR spectra (δ_H_ 0.0 to 6.5 ppm; δ_H_ 0.0 to 6.5 ppm) of centratherin. Figure S22: ^1^H NMR spectra (δ_H_ −0.5 to 7.0 ppm) of goyazensolide. Figure S23: ^13^C NMR spectra (δ_C_ 0.0 to 240.0 ppm) of goyazensolide. Figure S24: HSQC (^1^H-^13^C) NMR spectra (δ_H_ −0.25 to 6.5 ppm; δ_C_ 0 to 140 ppm) of goyazensolide. Figure S25: (^1^H-^1^H) COSY NMR spectra (δ_H_ 0.0 to 6.5 ppm; δ_H_ 1.0 to 7.5 ppm) of goyazensolide. Figure S26: (^1^H-^13^C) HMBC NMR spectra (δ_H_ −1.0 to 9.0 ppm; δ_C_ 0.0 to 210 ppm) of goyazensolide. Figure S27: (^1^H-^1^H) NOESY NMR spectra (δ_H_ −1.0 to 7.5 ppm; δ_H_ −0.5 to 7.0 ppm) of goyazensolide.
