# Supplementary material for: Isolation and In Vitro Activity of Sesquiterpene Lactones from Eremanthus crotonoides as SARS-CoV-2 Protease Inhibitors and Cytotoxic Agents
Source: Molecules. 2025 Oct 11;30(20):4053. doi: 10.3390/molecules30204053 (PMC12566191; doi:10.3390/molecules30204053)
Supplement: Supplementary file 1 [file molecules-30-04053-s001.zip › molecules-3830111-supplementary.pdf]

Supplementary Material

Isolation and In Vitro Activity of Sesquiterpene Lactones from *Eremanthus crotonoides* as SARS-CoV-2 Protease Inhibitors and Cytotoxic Agents

Patricia Homobono Brito de Moura [1,2], Natalie Giovanna da Rocha Ximenes [1], Carla Monteiro Leal [1], Beatriz Bastos Santos [1], Larissa Esteves Carvalho Constant [3], Stephany da Silva Costa [3], Shaft Corrêa Pinto [4], Michelle Frazao Muzitano [4], Diego Allonso [5], Ludger A. Wessjohann [2,*] and Ivana Correa Ramos Leal [1,*]

[1] Laboratory of Natural products and Biological Assays, Natural Products and Food Department, Pharmacy Faculty, Center of Health Sciences, Federal University of Rio de Janeiro (UFRJ), Rio de Janeiro 21941-902, RJ, Brazil

[2] Leibniz Institute of Plant Biochemistry, Department of Bioorganic Chemistry, Weinberg 3, 06120 Halle, Germany

[3] Laboratory of Biotechnology and Tissue Bioengineering, Institute of Biophysics Carlos Chagas Filho, Center of Health Sciences, Federal University of Rio de Janeiro (UFRJ), Rio de Janeiro 21941-902, RJ, Brazil

[4] Laboratory of Bioatives Products, Institute of Pharmaceutical Sciences, Federal University of Rio de Janeiro (UFRJ), Macaé 27933-378, RJ, Brazil

[5] Department of Pharmaceutical Biotechnology, Pharmacy Faculty, Center of Health Sciences, Federal University of Rio de Janeiro (UFRJ), Rio de Janeiro 21941-902, RJ, Brazil; diegoallonso@gmail.com

* Correspondence: wessjohann@ipb-halle.de (L.A.W.); ivanafarma@yahoo.com.br or ivana@pharma.ufrj.br (I.C.R.L.)

Summary

Tables

Table S1. Compounds characterized by UPLC-(ESI)-MS/MS in the crude extract of *Eremanthus crotonoides* compared to literature, GNPS (cosine similarity), and considering fragmentation patterns ……03

Table S2. MZmine 2.53 software parameters ……04

Table S3. EBEC, FDCM, centratherin and goyazensolide from the leaves of the *Eremanthus crotonoides* effects on the PLpro enzyme……05

Figures

Figure S1. Chlorogenic acid MS^2 spectrum *m/z* 355.1019 $[M+H]^+$……06

Figure S2. Caffeoylquinic acid (isomer I) MS^2 spectrum *m/z* 517.1345 $[M+H]^+$……06

Figure S3. Caffeoylquinic acid (isomer II) MS^2 spectrum *m/z* 517.1326 $[M+H]^+$……07

Figure S4. Quercetin-3-*O*-glucoside MS^2 spectrum *m/z* 465.1027 $[M+H]^+$……07

Figure S5. Caffeoylquinic acid (isomer III) MS^2 spectrum *m/z* 517.1337 $[M+H]^+$……08

Figure S6. Isorhamnetin-3-*O*-glucoside MS^2 spectrum *m/z* 479.1178 $[M+H]^+$……08

Figure S7. Tiliroside MS^2 spectrum *m/z* 595.1450 $[M+H]^+$……09

Figure S8. Luteolin MS^2 spectrum *m/z* 287.0565 $[M+H]^+$……09

Figure S9. Isorhamnetin MS^2 spectrum *m/z* 317.0670 $[M+H]^+$……10

Figure S10. Goiazensolido MS^2 spectrum *m/z* 361.1307 $[M+H]^+$……10

Figure S11. Budlein A isobutyrate MS^2 spectrum *m/z* 363.1448 $[M+H]^+$……………………………………..11

Figure S12. Centratherin MS^2 spectrum *m/z* 375.1440 $[M+H]^+$…………………………………………….11

Figure S13. Overlaped ^1H NMR spectra (δ_H -0.5 to 8.0 ppm) of the crude extract and the dichloromethane fraction of *E. crotonoides*……………………………………………………………………………………12

Figure S14. HPLC-DAD of dichloromethane fraction monitored at λ = 269 and the UV spectra of their six peaks collected………………………………………………………………………………………………..12

Figure S15. ^1H NMR spectra (δ_H -0.25 to 6.5 ppm) of goyazensolide…………………………………...13

Figure S16. ^{13}C NMR spectra (δ_C 0.0 to 210.0 ppm) of goyazensolide…………………………………..13

Figure S17. HSQC (^1H-^{13}C) NMR spectra (δ_H -0.25 to 6.5 ppm; δ_C 10 to 145 ppm) of centratherin………14

Figure S18. HMBC (^1H-^{13}C) NMR spectra (δ_H 1.2 to 6.6 ppm; δ_C 0.0 to 210 ppm) of centratherin……….14

Figure S19. (^1H-^1H) NOESY NMR spectra (δ_H 0.0 to 6.5 ppm; δ_H 0.0 to 7.0 ppm) of centratherin………..15

Figure S20. (^1H-^1H) COSY NMR spectra (δ_H 0.0 to 7.0 ppm; δ_H 0.0 to 6.5 ppm) of centratherin………….15

Figure S21. (^1H-^1H) TOCSY NMR spectra (δ_H 0.0 to 6.5 ppm; δ_H 0.0 to 6.5 ppm) of centratherin………..16

Figure S22. ^1H NMR spectra (δ_H -0.5 to 7.0 ppm) of goyazensolide………………………………………17

Figure S23. ^{13}C NMR spectra (δ_C 0.0 to 240.0 ppm) of goyazensolide……………………………………17

Figure S24. HSQC (^1H-^{13}C) NMR spectra (δ_H -0.25 to 6.5 ppm; δ_C 0 to 140 ppm) of goyazensolide……..18

Figure S25. (^1H-^1H) COSY NMR spectra (δ_H 0.0 to 6.5 ppm; δ_H 1.0 to 7.5 ppm) of goyazensolide……….18

Figure S26. (^1H-^{13}C) HMBC NMR spectra (δ_H -1.0 to 9.0 ppm; δ_C 0.0 to 210 ppm) of goyazensolide…….19

Figure S27. (^1H-^1H) NOESY NMR spectra (δ_H -1.0 to 7.5 ppm; δ_H -0.5 to 7.0 ppm) of goyazensolide…...19

Table S1. Compounds characterized by UPLC-(ESI)-MS/MS in the crude extract of *Eremanthus crotonoides* compared to literature, GNPS (cosine similarity), and considering fragmentation patterns.

	Putative identification	RT	Parent Ion	MS^2 fragmentation	Cosine**
1	Chlorogenic acid	3.53	355.1019 $[M+H]^+$	**163.0376***, 145.0270, 135.0428, 117.0322, 89.0376	0.99
2a	Caffeoylquinic acid (isomer I)	4.26	517.1345 $[M+H]^+$	**163.0388***, 145.0268, 135.0419, 117.0331, 89.0374	0.98
2b	Caffeoylquinic acid (isomer II)	5.66	517.1326 $[M+H]^+$	**163.0378***, 145.0267, 135.0440, 89.0374	0.98
3	Quercetin-3-*O*-glucoside	5.88	465.1027 $[M+H]^+$	**303.0490***	NF
2c	Caffeoylquinic acid (isomer III)	6.01	517.1337 $[M+H]^+$	**163.0378***, 145.0270, 135.0424, 117.0322, 89.0378	0.98
4	Isorhamnetin-3-*O*-glucoside	6.45	479.1178 $[M+H]^+$	**317.0646***	0.95
5	Tiliroside	7.69	595.1450 $[M+H]^+$	309.0968, 287.0551, **147.0437***	0.97
6	Luteolin	7.78	287.0565 $[M+H]^+$	269.0429, 241.0481, 213.0520, **153.0166***	0.93
7	Isorhamnetin	8.15	317.0670 $[M+H]^+$	302.0404, 285.0369, **274.0450***, 257.0422, 245.0425, 228.0398, 137.0219	0.94
8	Goyazensolide	8.60	361.1307 $[M+H]^+$	343.1219, 297.1161, 275.0948, 257.0832, **229.0885***, 183.0820, 155.0869, 128.0631, 91.0546	0.73
9	Budlein A isobutyrate	8.68	363.1448 $[M+H]^+$	345.1365, 293.1039, 275.0927, 257.0825, **229.0875***, 183.0816, 155.0856, 128.0620, 91.0542	NF
10	Centratherin	9.20	375.1440 $[M+H]^+$	357.1305, 329.8387, 311.8253, 257.0825, **229.0867***, 183.0815, 155.0856, 129.0696, 83.0489	NF

*Base peaks are marked in bold

**Cosine available in: https://gnps.ucsd.edu/ProteoSAFe/status.jsp?task=4f12c4f9a8f640dcb32c1633ded31735

NF: no cosine value because no spectral match in molecular library search in GNPS

Table S2. MZmine 2.53 software parameters.

	**Negative ionization mode**	**Positive ionization mode**
**Mass detection**	Noise level MS^1: 1.5E3<br>Noise level MS^2: 1.5E2	Noise level MS^1: 1.0E3<br>Noise level MS^2: 1.0E2
**ADAP Chromatogram builder**	Min group size: 5<br>Group intensity threshold: 2.4E4<br>Min highest intensity: 4.4E4<br>*m/z* tolerance: 0.01 or 10 ppm	Min group size: 5<br>Group intensity threshold: 3.4E4<br>Min highest intensity: 1.0E5<br>*m/z* tolerance: 0.01 or 10 ppm
**Chromatogram deconvolution**	Algorithm: Local minimum search<br>Chromatographic threshold: 5 %<br>Search minimum in RT range (min): 0.5<br>Minimum relative height: 1%<br>Minimum absolute height: 1.0E2<br>Min ratio of peak top/edge: 1<br>Peak duration range (min): 0-10	
**Isotopic peaks grouper**	*m/z* tolerance: 0.01 or 10 ppm<br>Retention time tolerance: 2 %<br>Maximum charge: 2	
**Alignment**	Join aligner<br>*m/z* tolerance: 0.01 or 10 ppm<br>Weight for *m/z*: 75<br>Retention time tolerance: 2 %<br>Weight for RT: 25	

Table S3. EBEC, FDCM, centratherin and goyazensolide from the leaves of the *Eremanthus crotonoides* effects on the rPLpro enzyme.

Sample	Maximum inhibition conc. (µg/mL)	Maximum inhibition (%)	Apparent IC_{50} (µg/mL)
		**PLpro**	
**Crude Extract (EBEC)**	1,000 µg/mL	99.82 ± 0.005%	129.9 µg/mL
**Dichloromethane Fraction (FDCM)**	1,000 µg/mL	99.83 ± 0.03%	29.88 µg/mL
**Centratherin**	1,000 µM	96.77 ± 0.3%	11.90 µM
**Goyazensolide**	1,000 µM	80.73 ± 5.9%	317 µM
		**3CLpro**	
**Crude Extract (EBEC)**	1,000 µg/mL	88.10 ± 11.82	304 µg/mL
**Dichloromethane Fraction (FDCM)**	1,000 µg/mL	95.21 ± 1.32	10.27 µg/mL
**Centratherin**	1,000 µM	89.55 ± 0.97	164.8 µM
**Goyazensolide**	1,000 µM	96.14 ± 0.01	71.1 µM

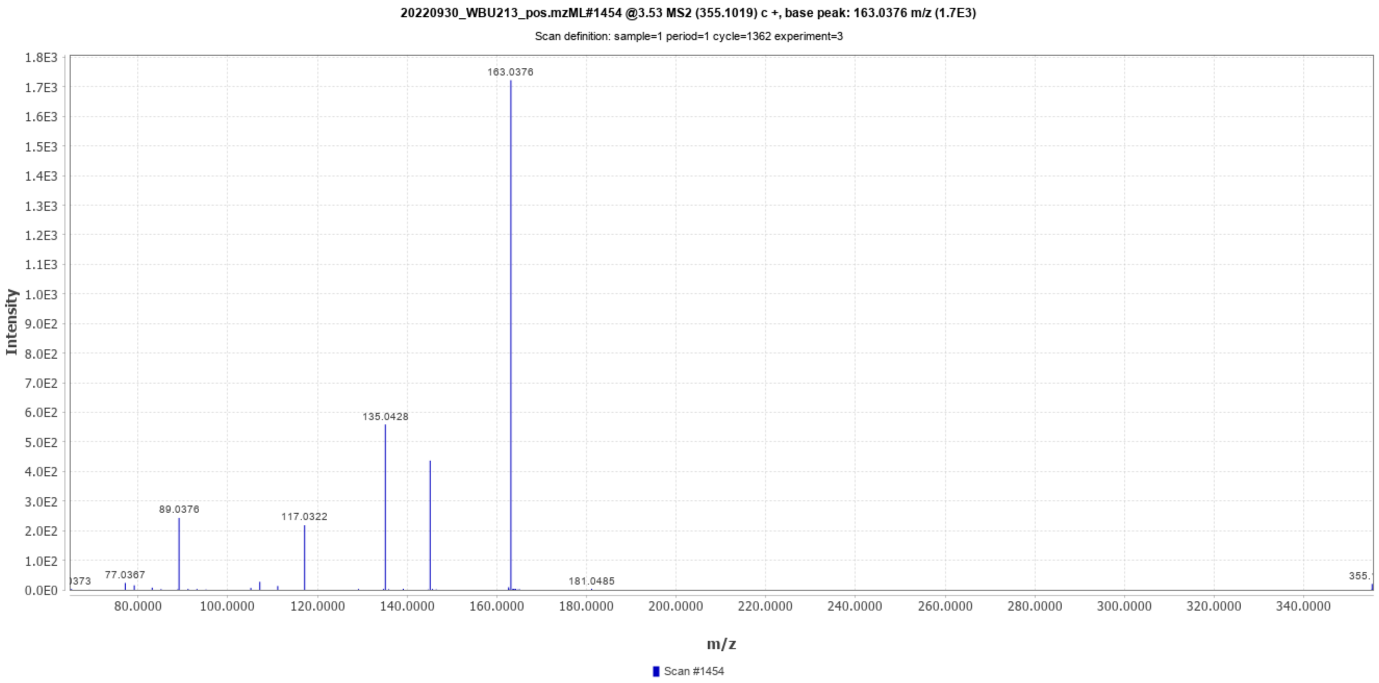

Figure S1. Chlorogenic acid MS^2 spectrum *m/z* 355.1019 $[M+H]^+$.

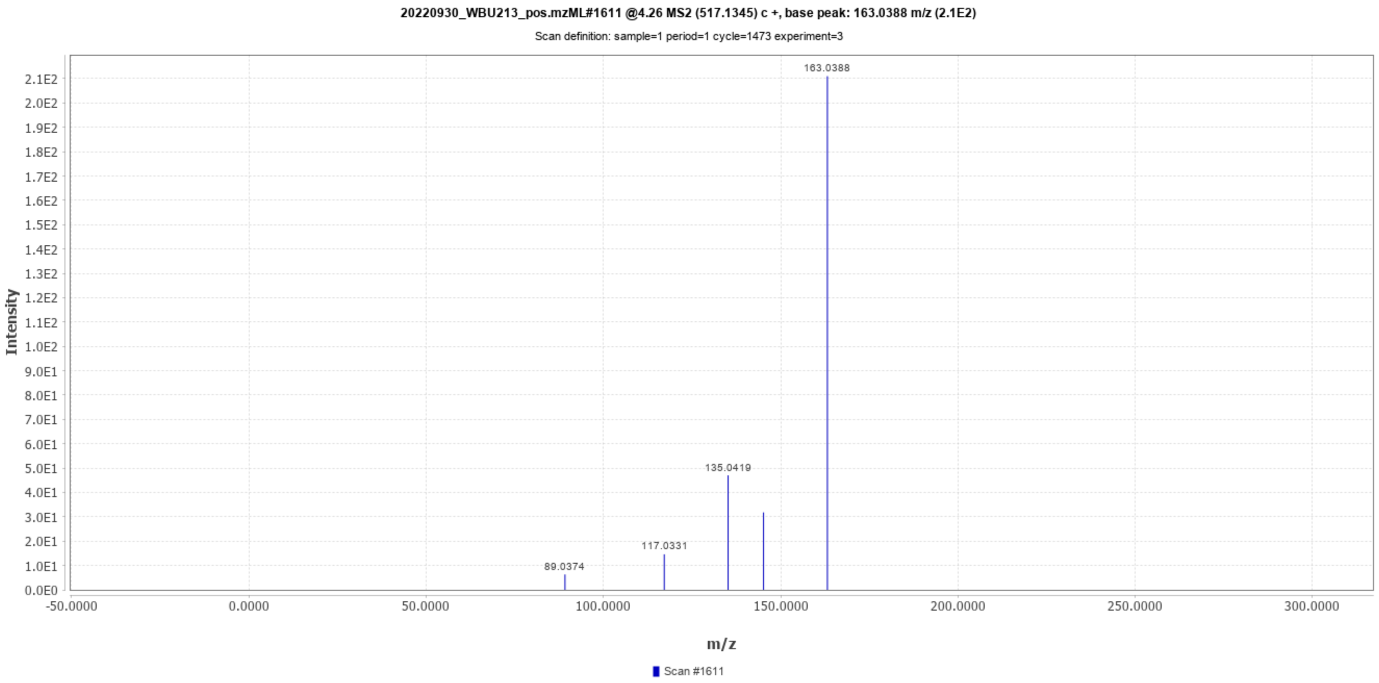

Figure S2. Caffeoylquinic acid (isomer I) MS^2 spectrum *m/z* 517.1345 $[M+H]^+$.

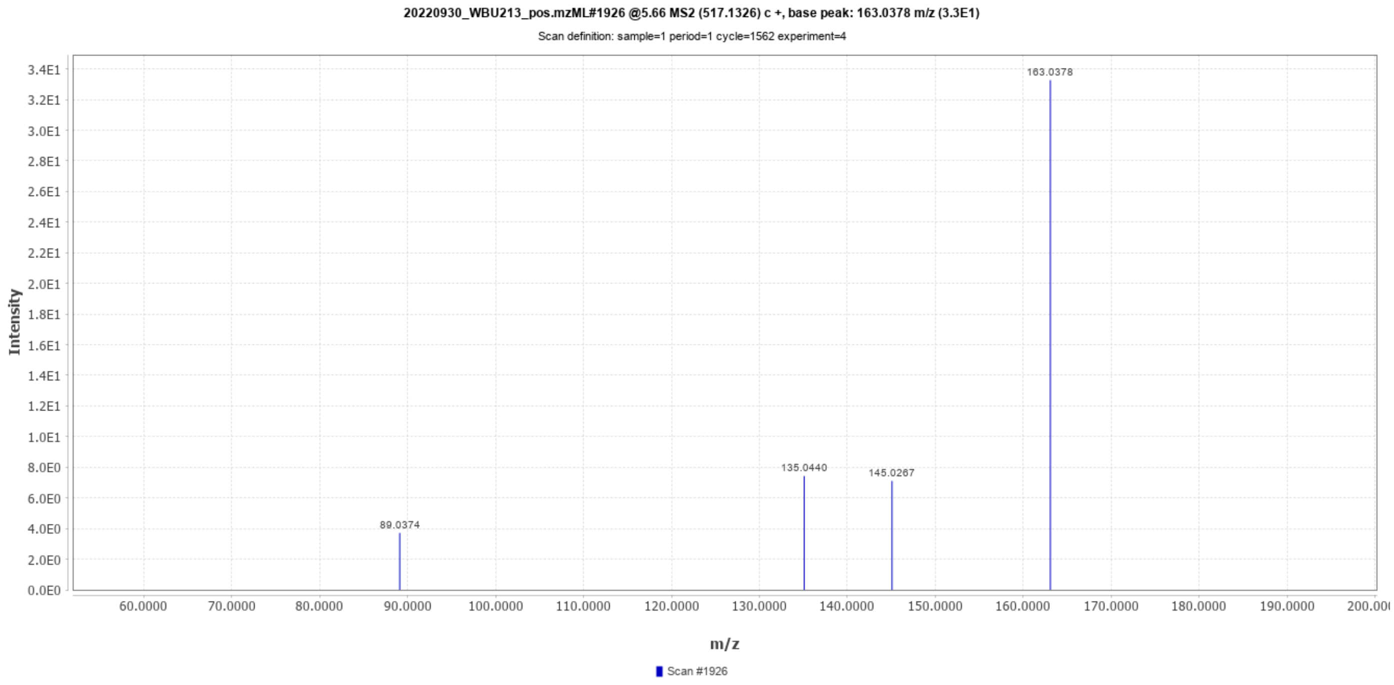

Figure S3. Caffeoylquinic acid (isomer II) MS^2 spectrum *m/z* 517.1326 $[M+H]^+$.

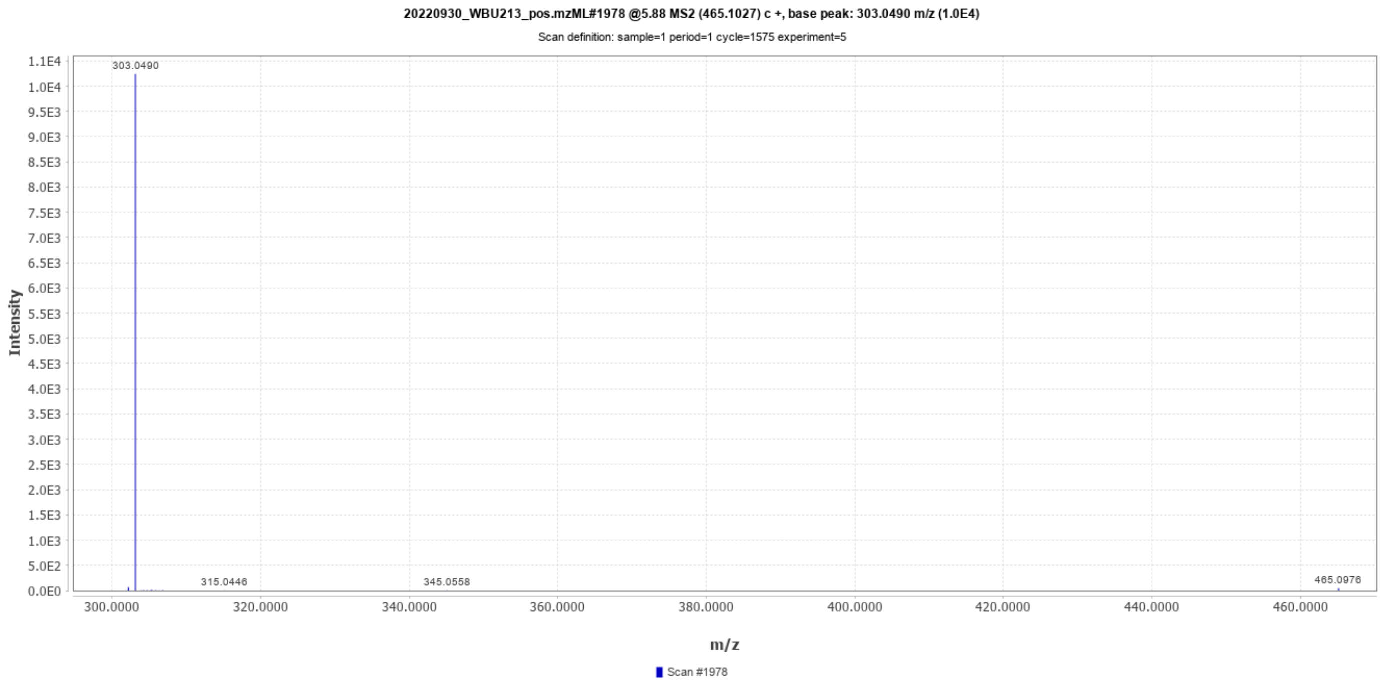

Figure S4. Quercetin-3-*O*-glucoside MS^2 spectrum *m/z* 465.1027 $[M+H]^+$.

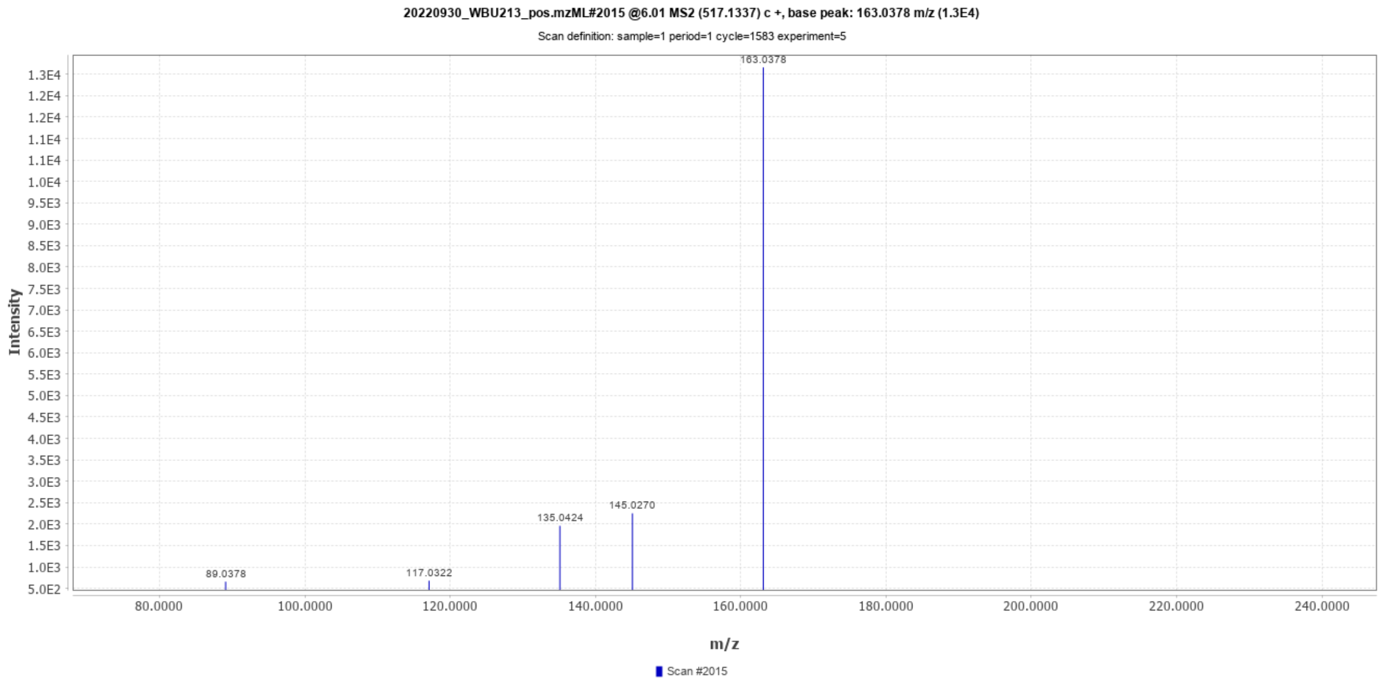

Figure S5. Caffeoylquinic acid (isomer III) MS^2 spectrum *m/z* 517.1337 $[M+H]^+$.

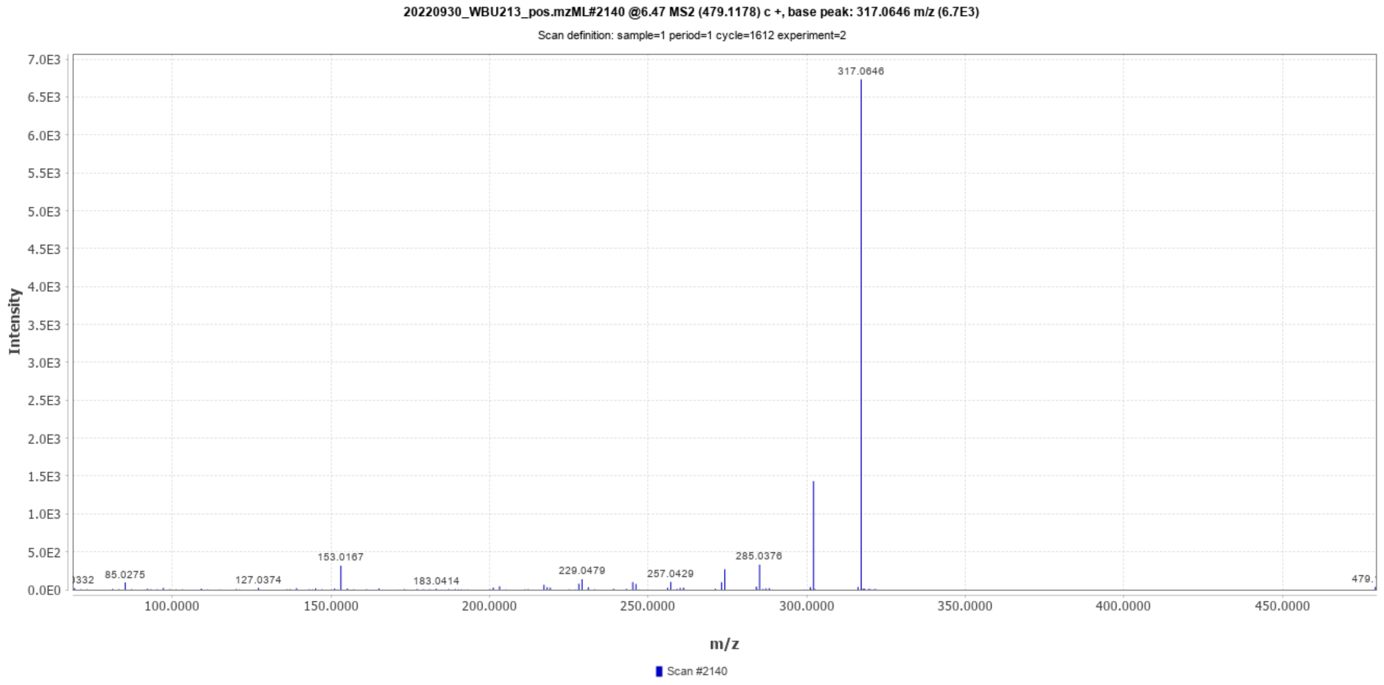

Figure S6. Isorhamnetin-3-*O*-glucoside MS^2 spectrum *m/z* 479.1178 $[M+H]^+$.

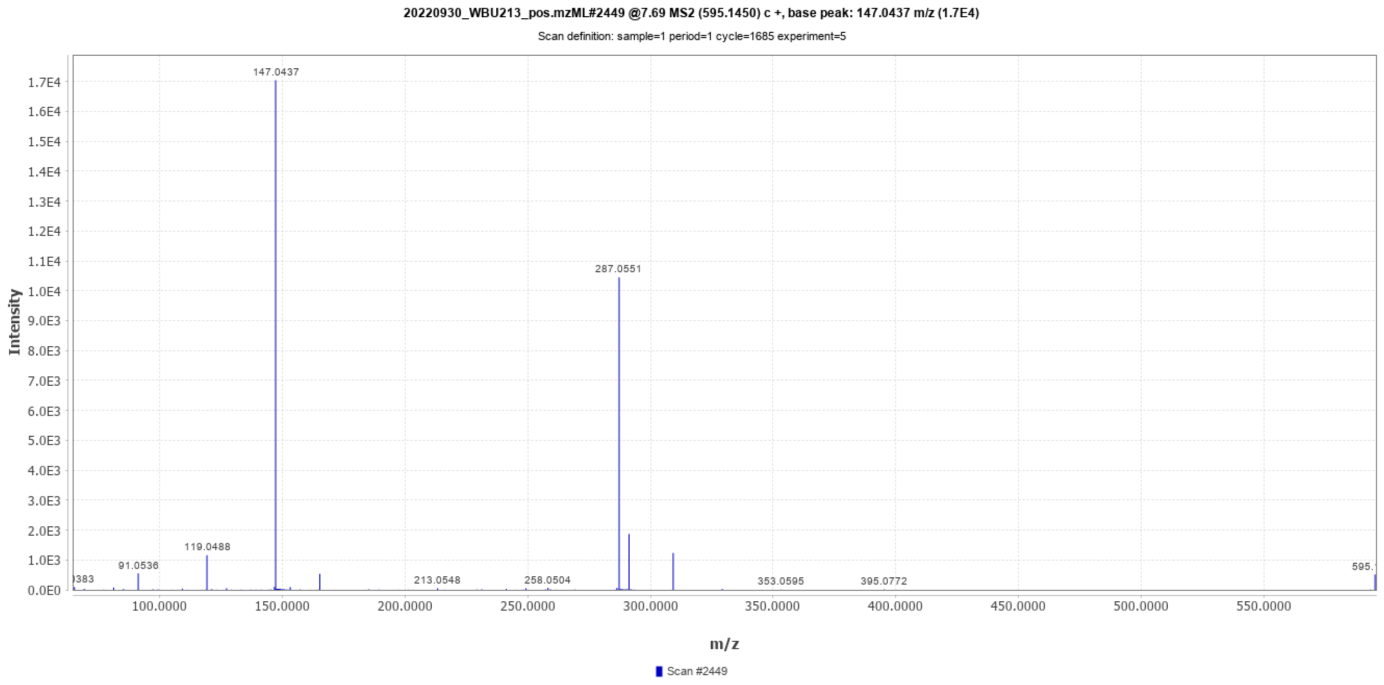

Figure S7. Tiliroside MS^2 spectrum *m/z* 595.1450 $[M+H]^+$.

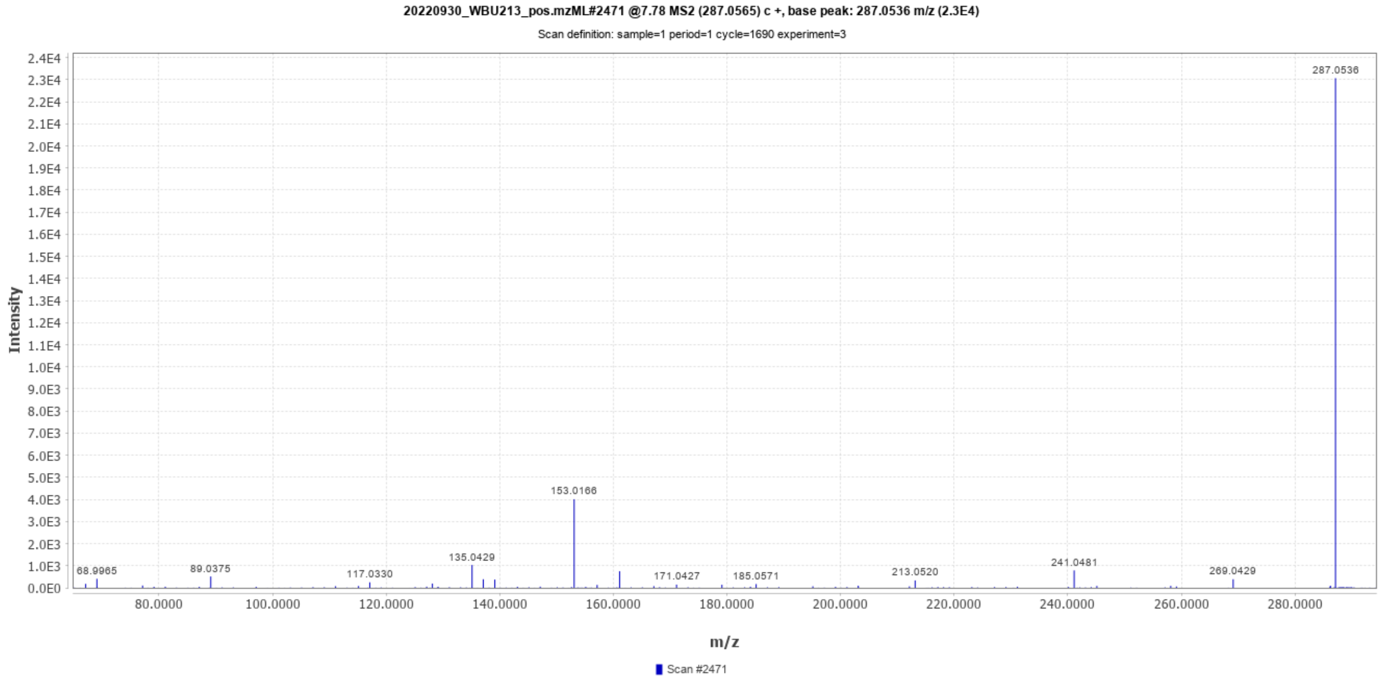

Figure S8. Luteolin MS^2 spectrum *m/z* 287.0565 $[M+H]^+$.

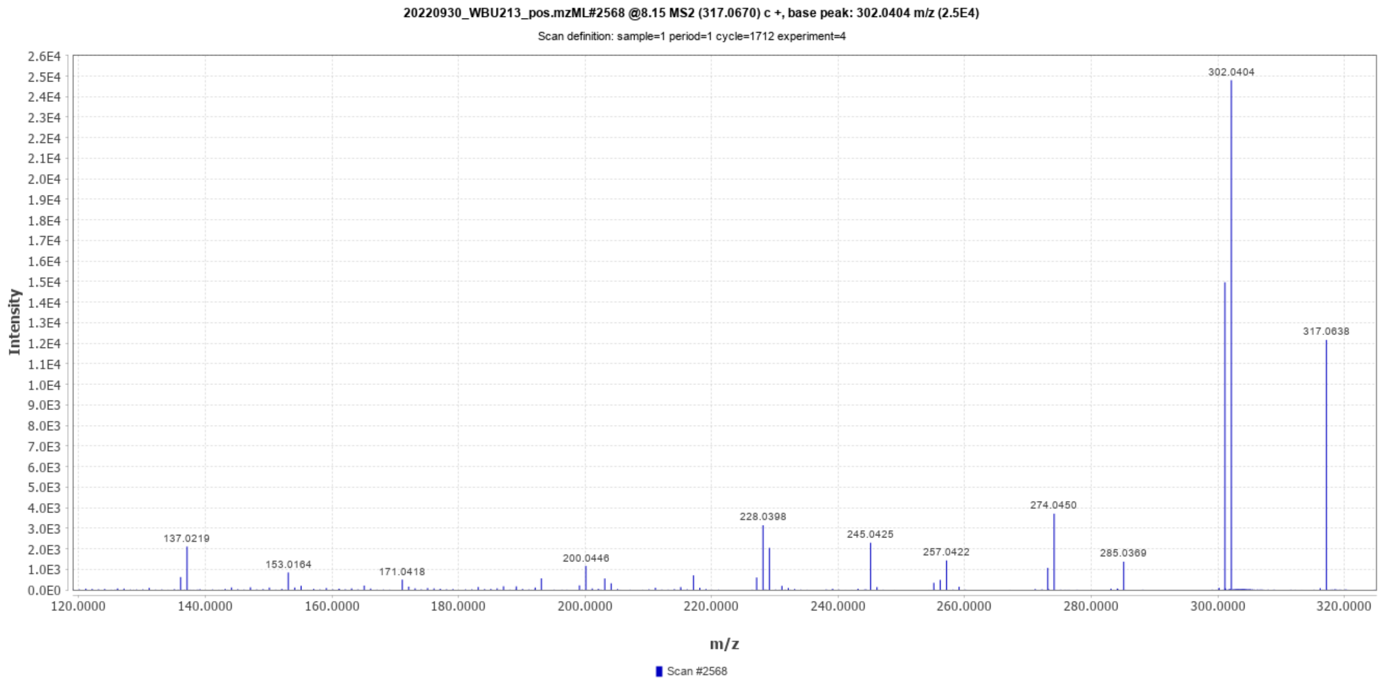

Figure S9. Isorhamnetin MS^2 spectrum *m/z* 317.0670 $[M+H]^+$.

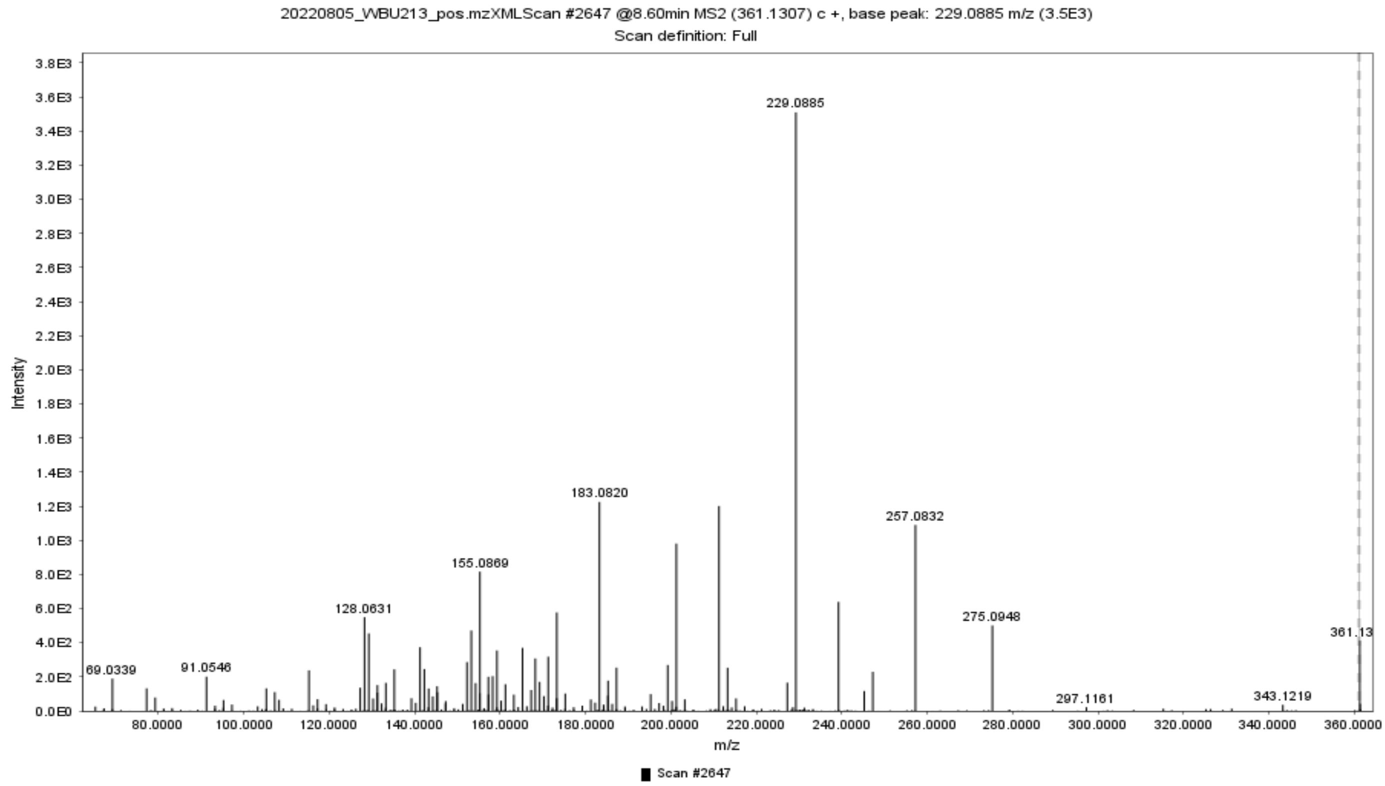

Figure S10. Goiazensolido MS^2 spectrum *m/z* 361.1307 $[M+H]^+$.

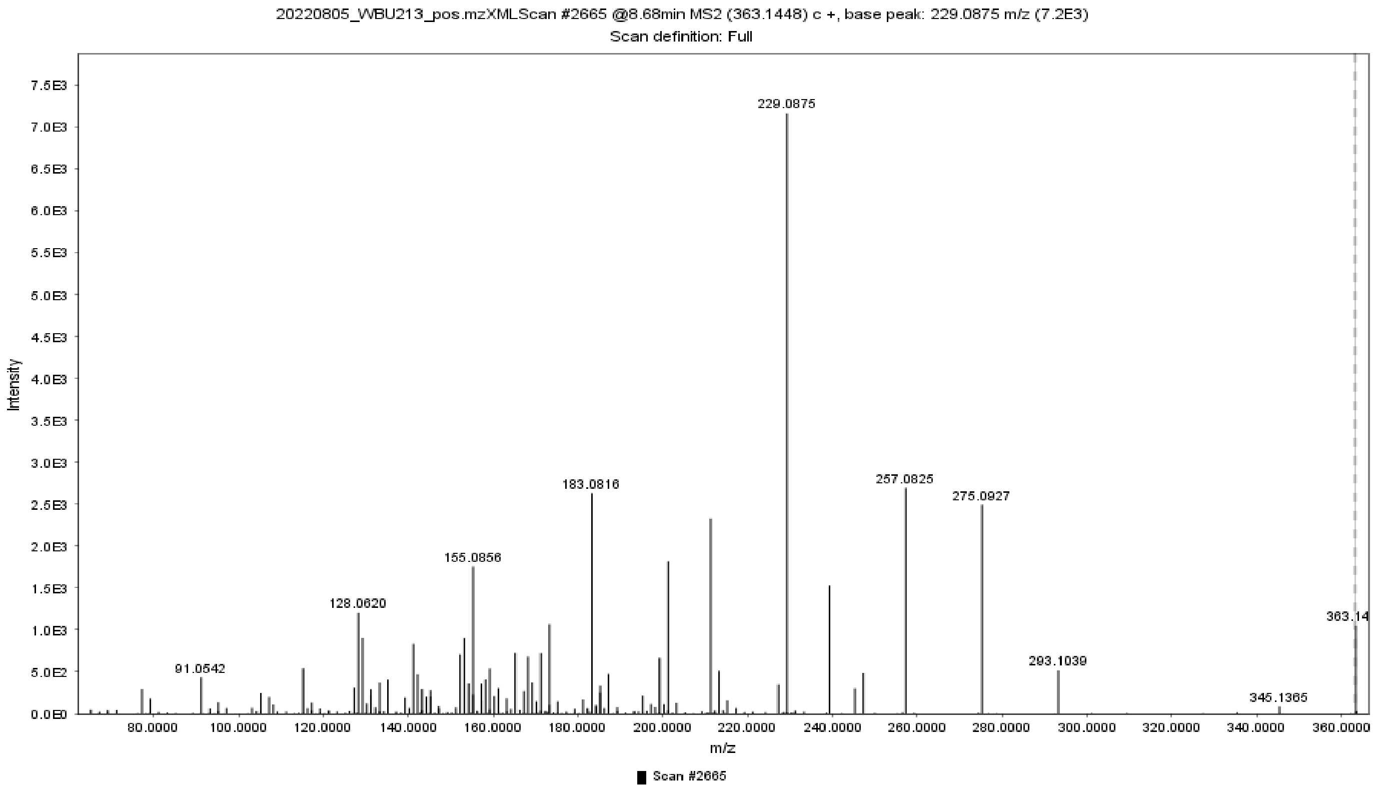

Figure S11. Budlein A isobutyrate MS^2 spectrum *m/z* 363.1448 $[M+H]^+$.

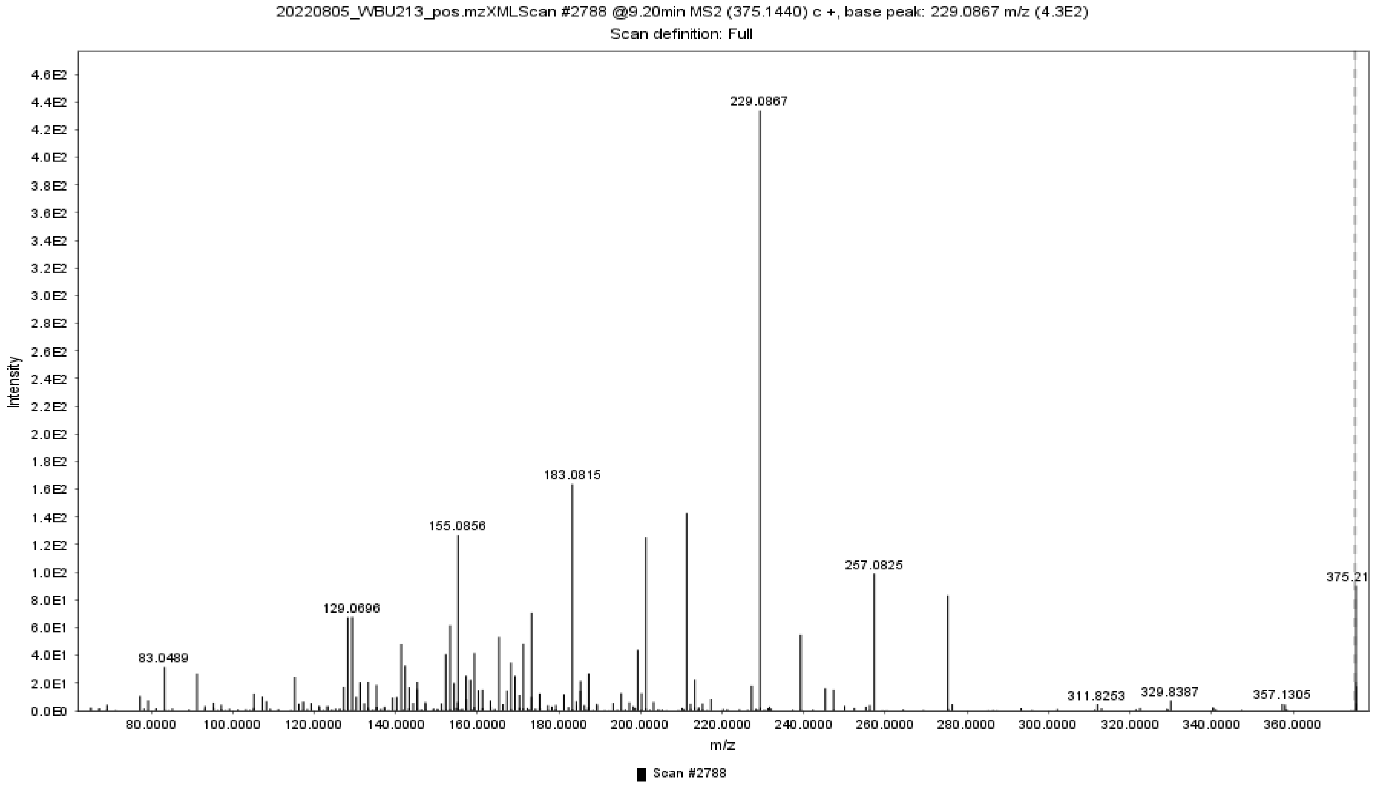

Figure S12. Centratherin MS^2 spectrum *m/z* 375.1440 $[M+H]^+$.

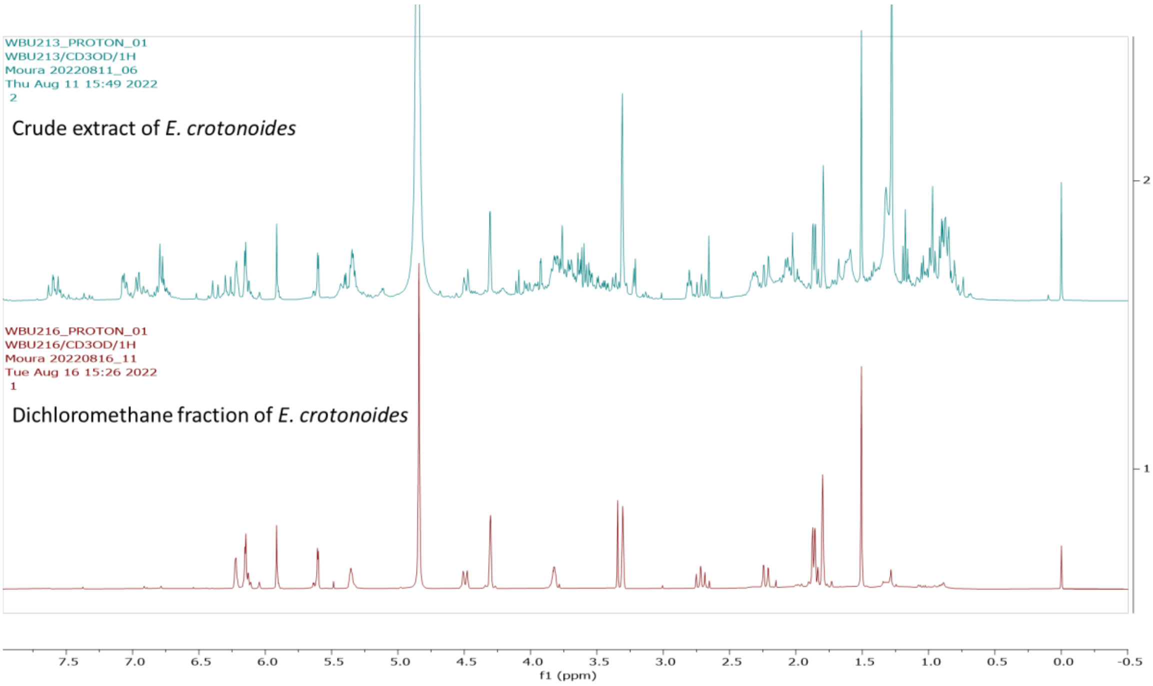

Figure S13. Overlaped ^{1}H NMR spectra (δ_H -0.5 to 8.0 ppm) of the crude extract and the dichloromethane fraction of *E. crotonoides*.

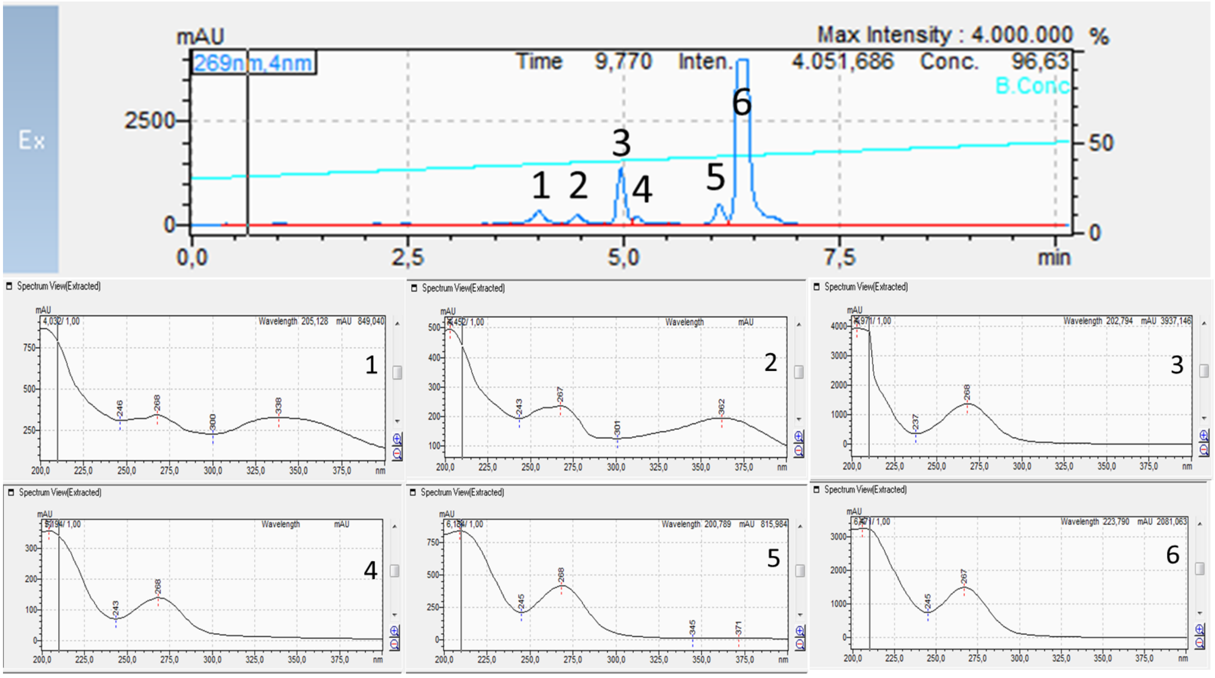

Figure S14. HPLC-DAD of dichloromethane fraction monitored at λ = 269 and the UV spectra of their six peaks collected.

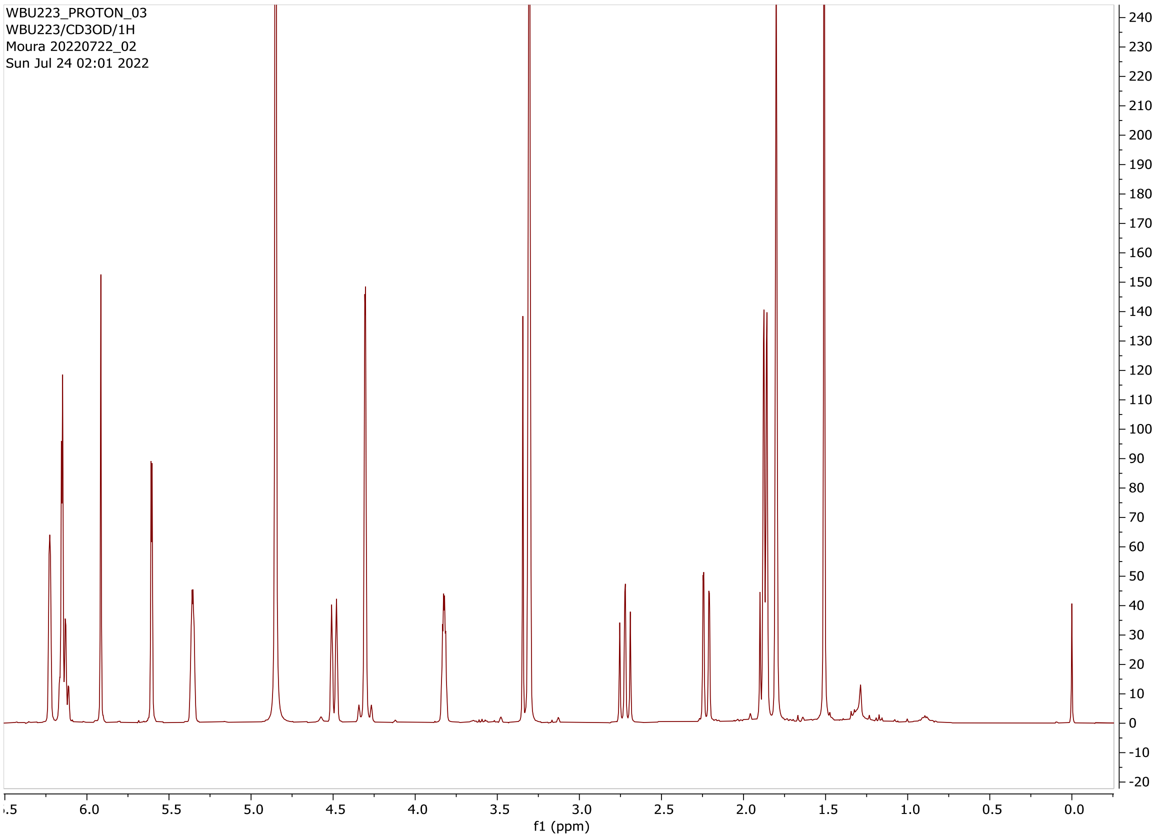

Figure S15. ^{1}H NMR spectra (δ_H -0.25 to 6.5 ppm) of goyazensolide.

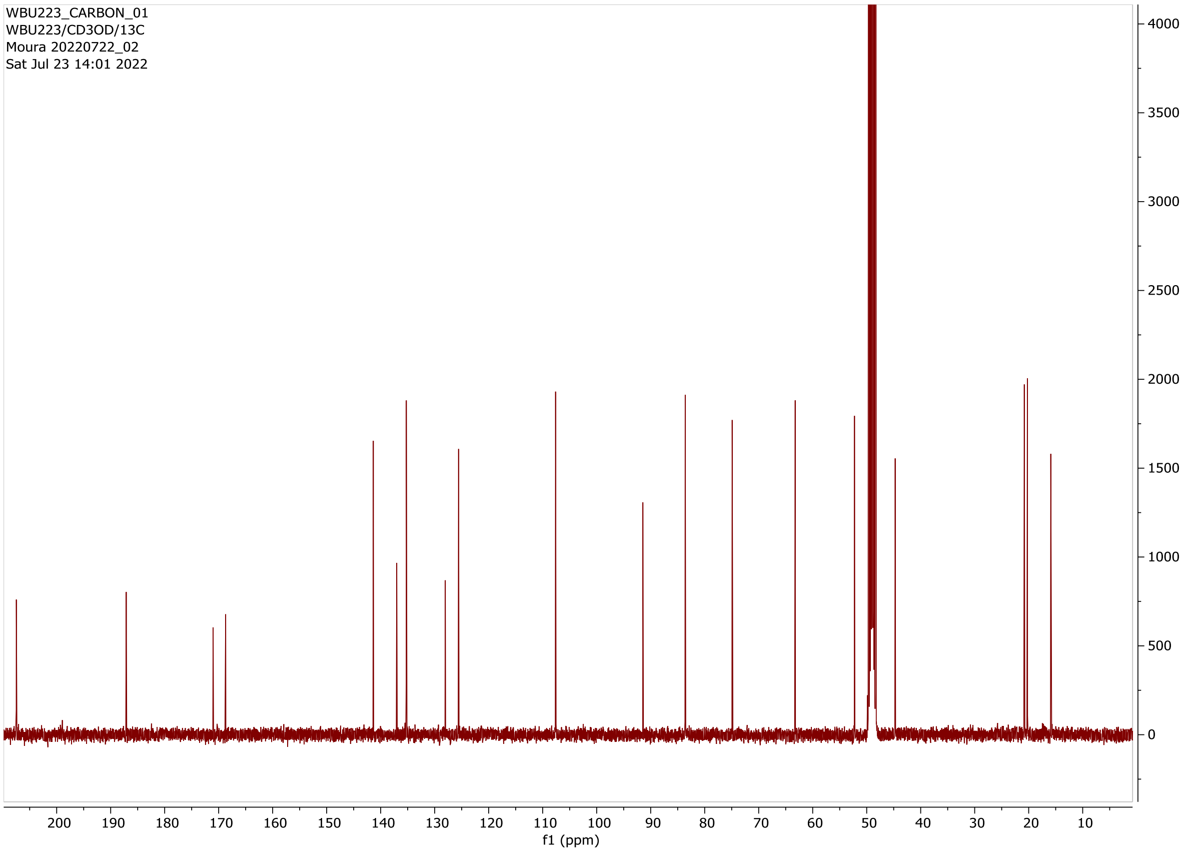

Figure S16. ^{13}C NMR spectra (δ_C 0.0 to 210.0 ppm) of goyazensolide.

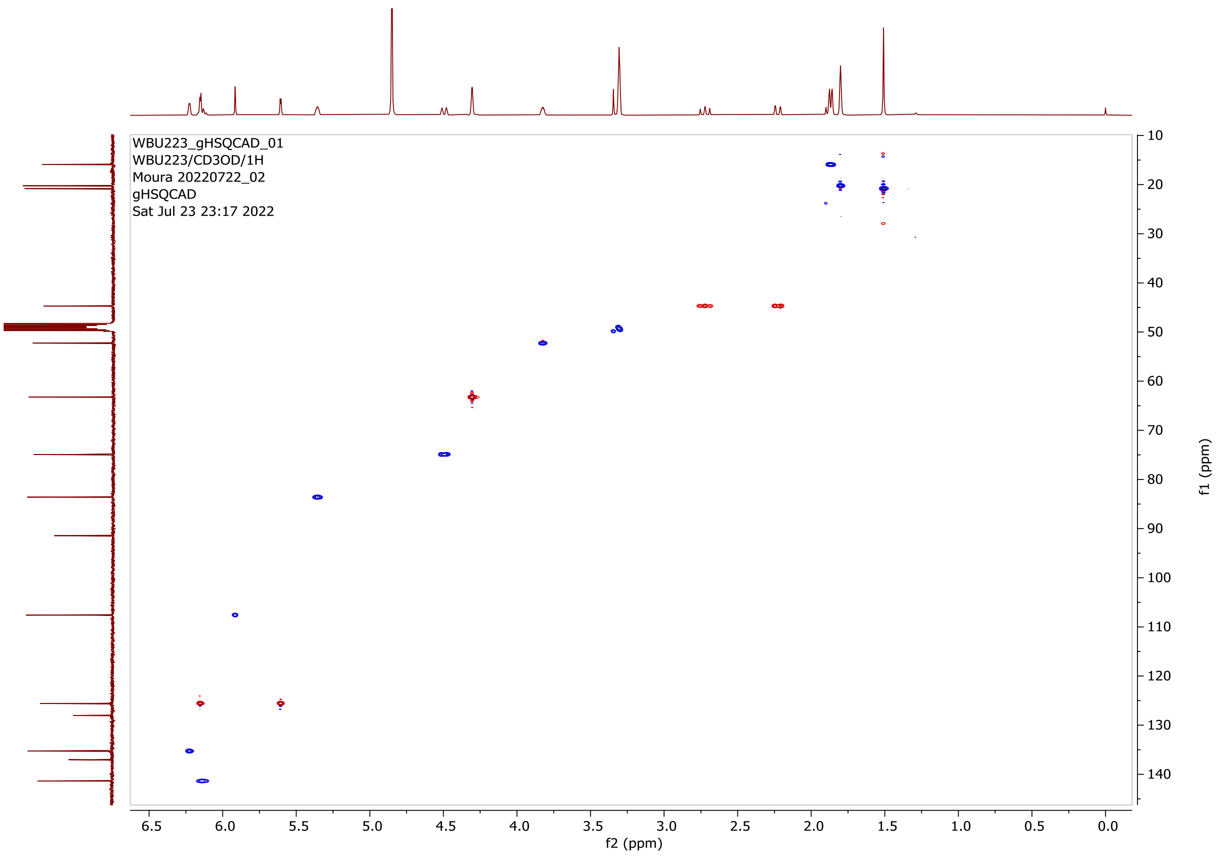

Figure S17. HSQC (^1H-^{13}C) NMR spectra (δ_H -0.25 to 6.5 ppm; δ_C 10 to 145 ppm) of centratherin.

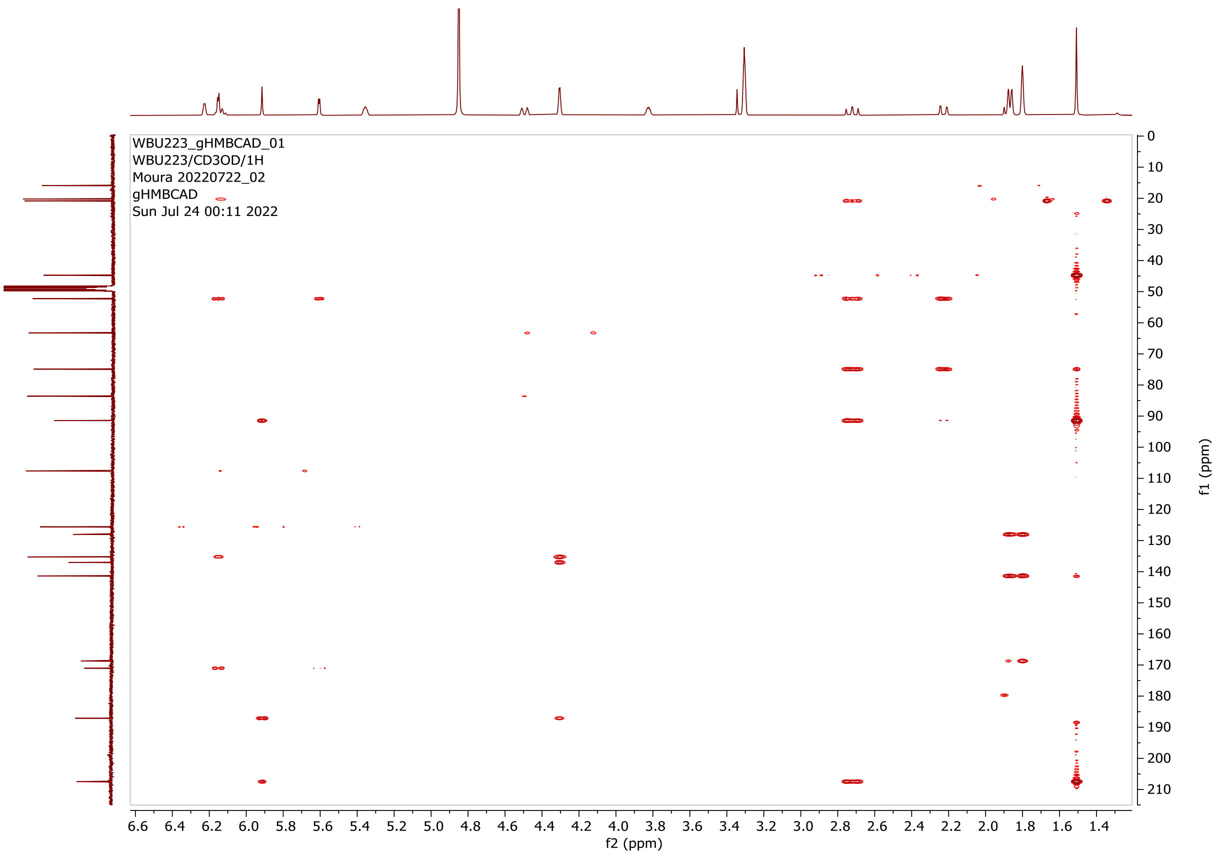

Figure S18. HMBC (^1H-^{13}C) NMR spectra (δ_H 1.2 to 6.6 ppm; δ_C 0.0 to 210 ppm) of centratherin.

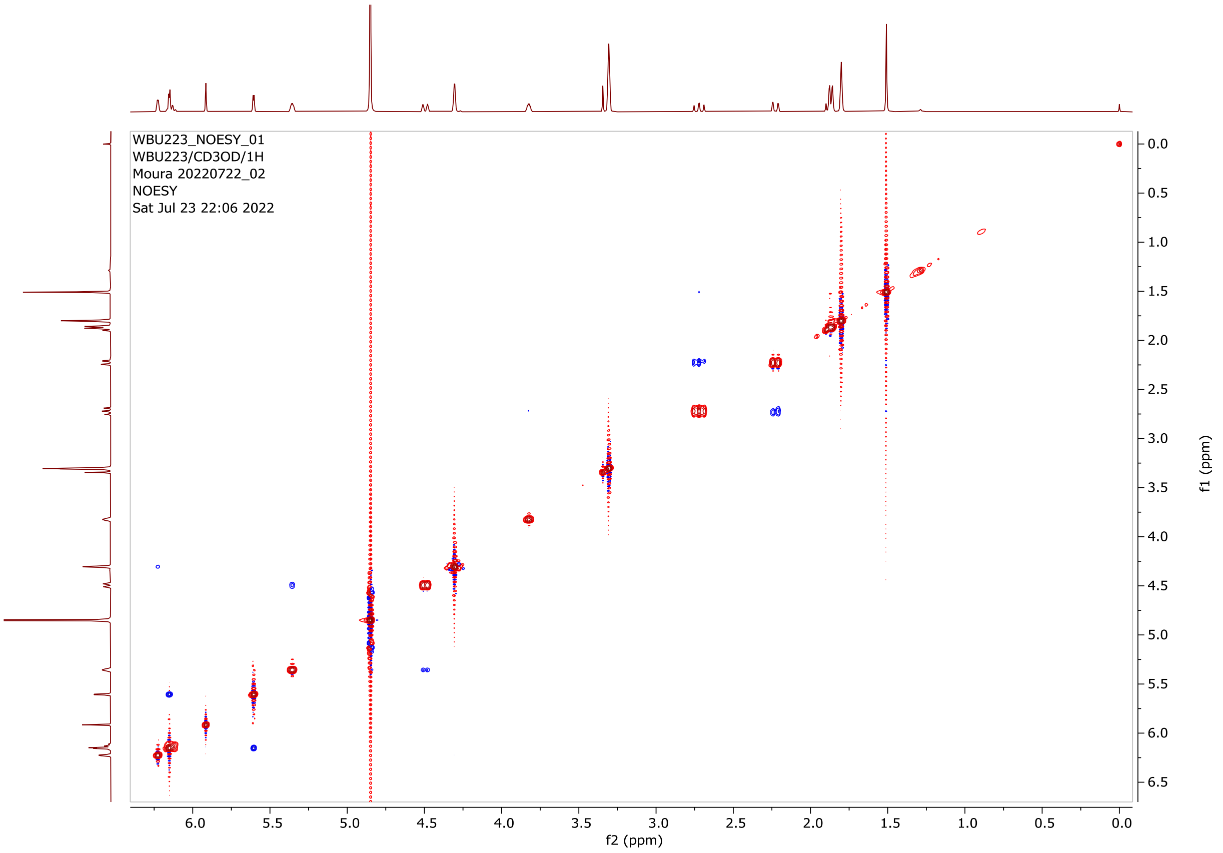

Figure S19. (^{1}H-^{1}H) NOESY NMR spectra (δ$_H$ 0.0 to 6.5 ppm; δ$_H$ 0.0 to 7.0 ppm) of centratherin.

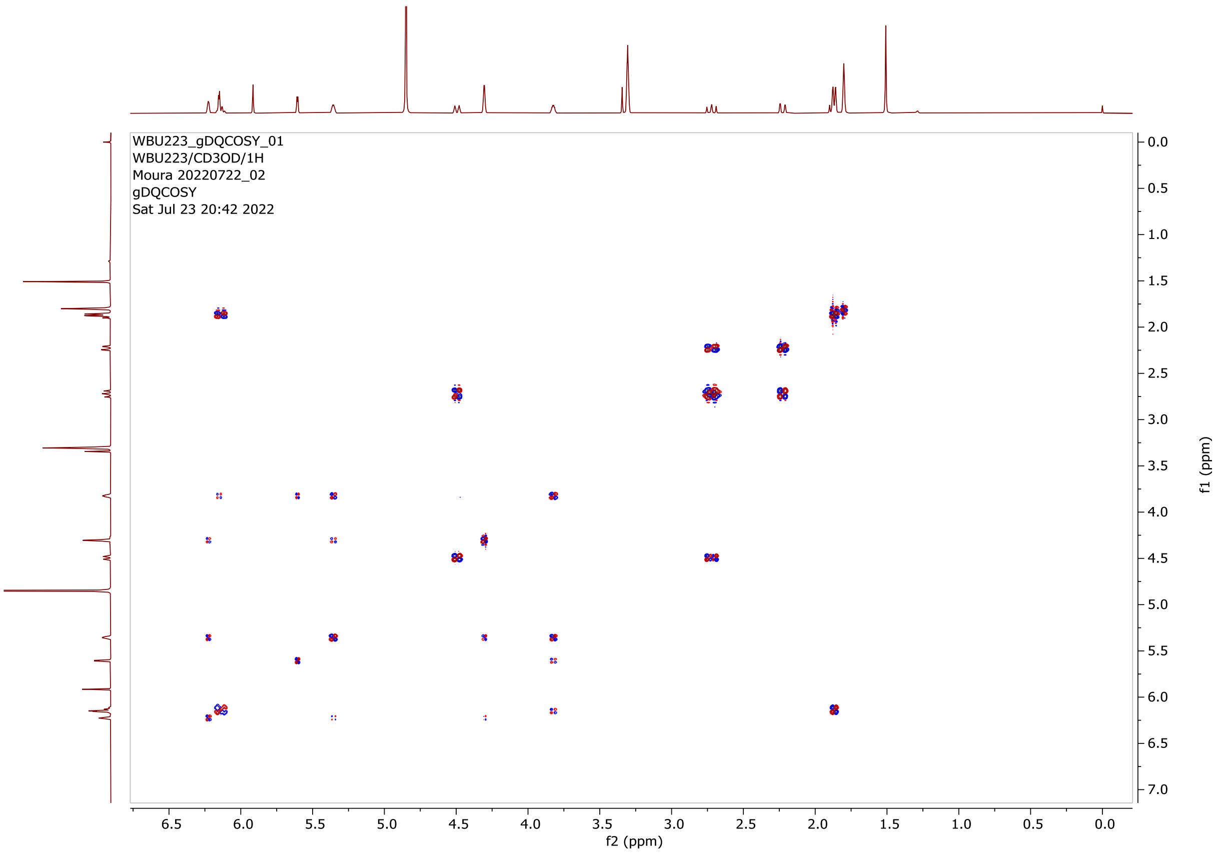

Figure S20. (^{1}H-^{1}H) COSY NMR spectra (δ$_H$ 0.0 to 7.0 ppm; δ$_H$ 0.0 to 6.5 ppm) of centratherin.

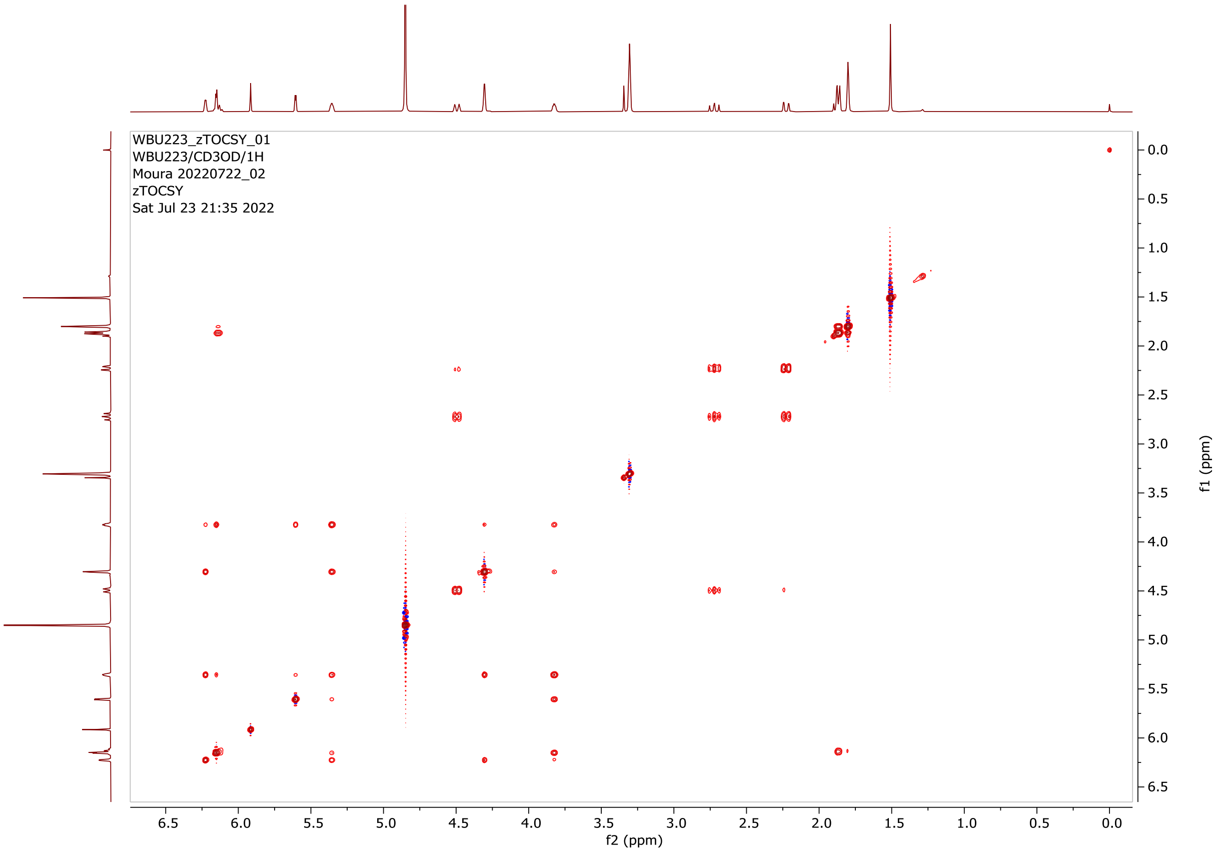

Figure S21. (^{1}H-^{1}H) TOCSY NMR spectra (δ_H 0.0 to 6.5 ppm; δ_H 0.0 to 6.5 ppm) of centratherin.

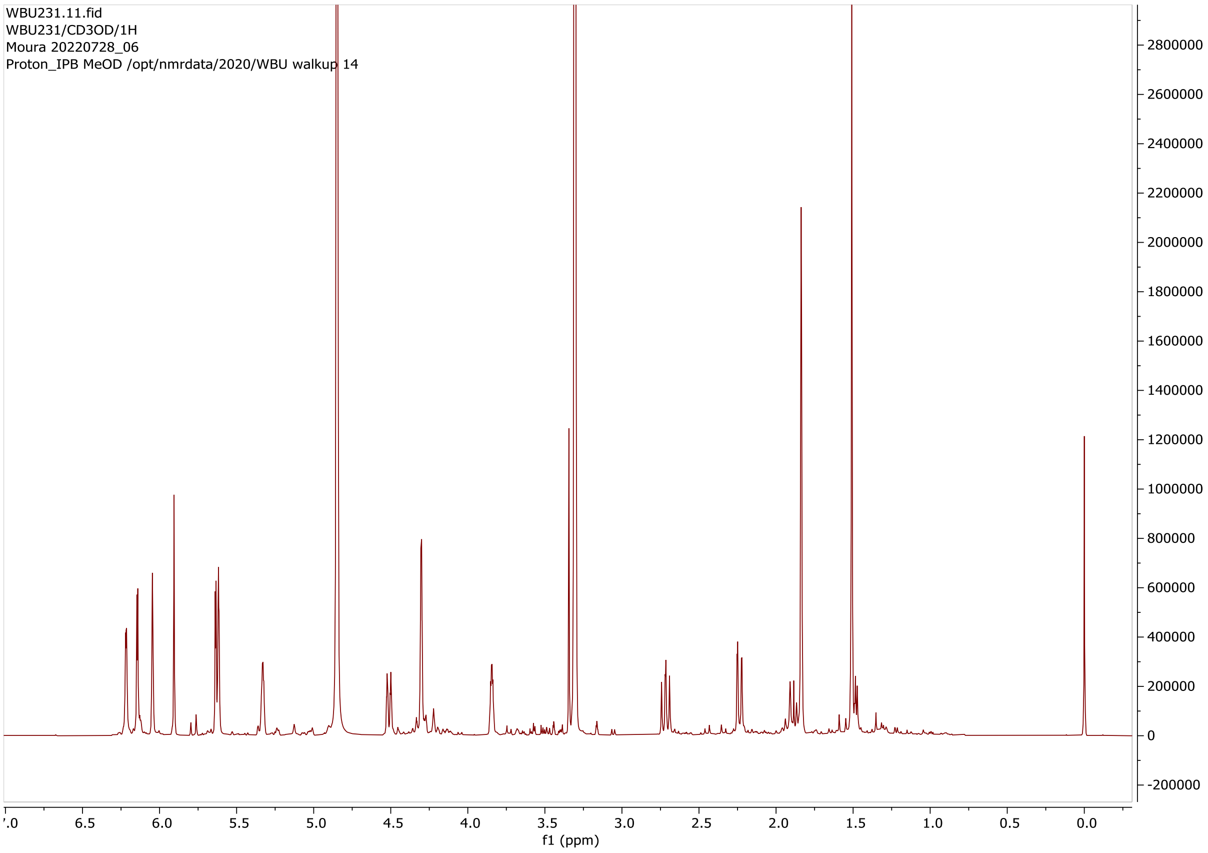

Figure S22. ^{1}H NMR spectra (δ_H -0.5 to 7.0 ppm) of goyazensolide.

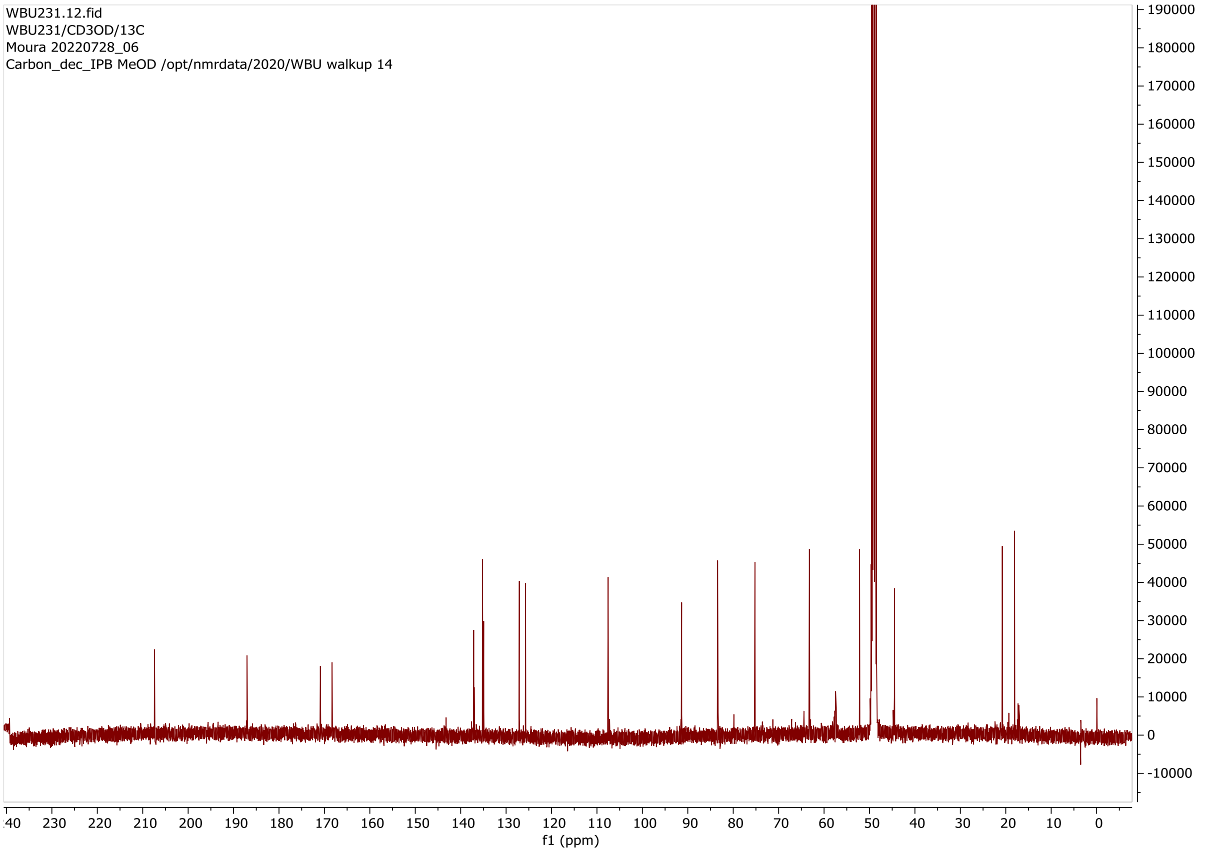

Figure S23. ^{13}C NMR spectra (δ_C 0.0 to 240.0 ppm) of goyazensolide.

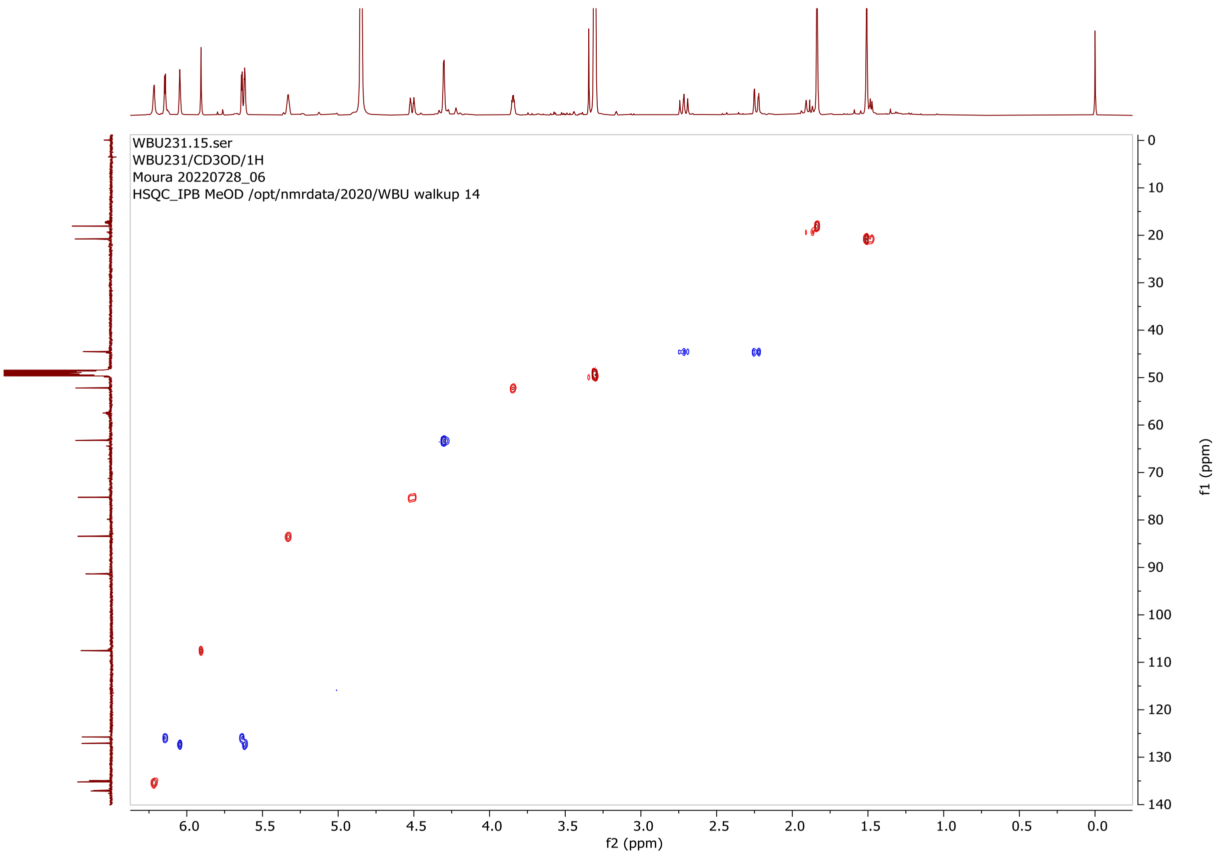

Figure S24. HSQC (^{1}H-^{13}C) NMR spectra (δ_H -0.25 to 6.5 ppm; δ_C 0 to 140 ppm) of goyazensolide.

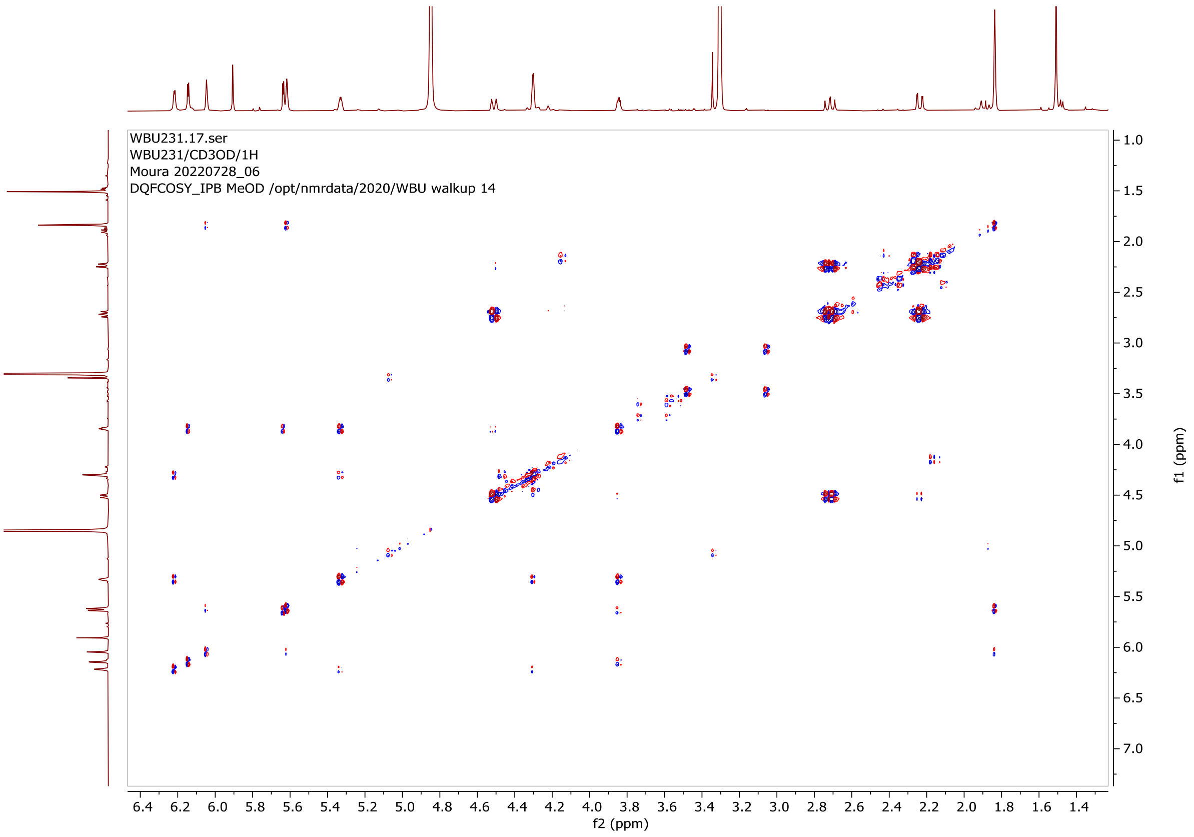

Figure S25. (^{1}H-^{1}H) COSY NMR spectra (δ_H 0.0 to 6.5 ppm; δ_H 1.0 to 7.5 ppm) of goyazensolide.

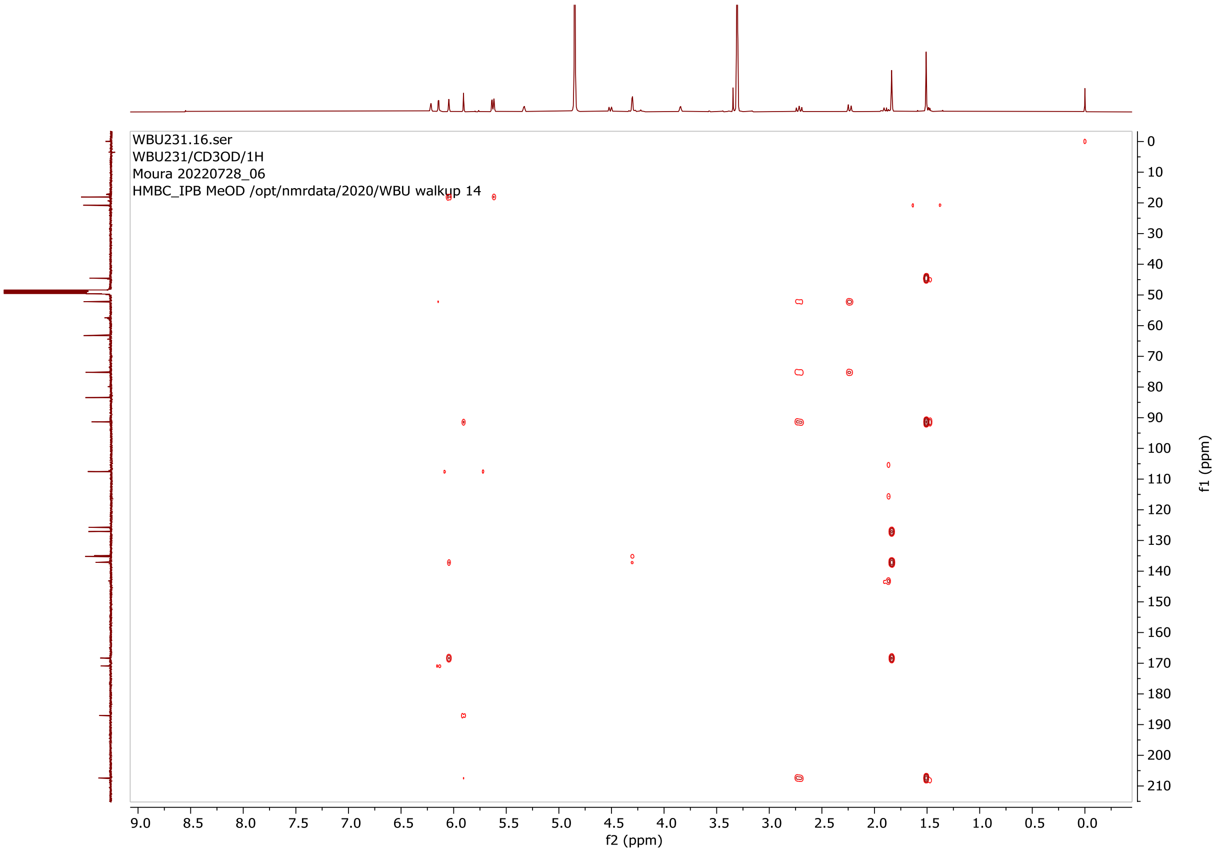

Figure S26. (^{1}H-^{13}C) HMBC NMR spectra (δ_H -1.0 to 9.0 ppm; δ_C 0.0 to 210 ppm) of goyazensolide.

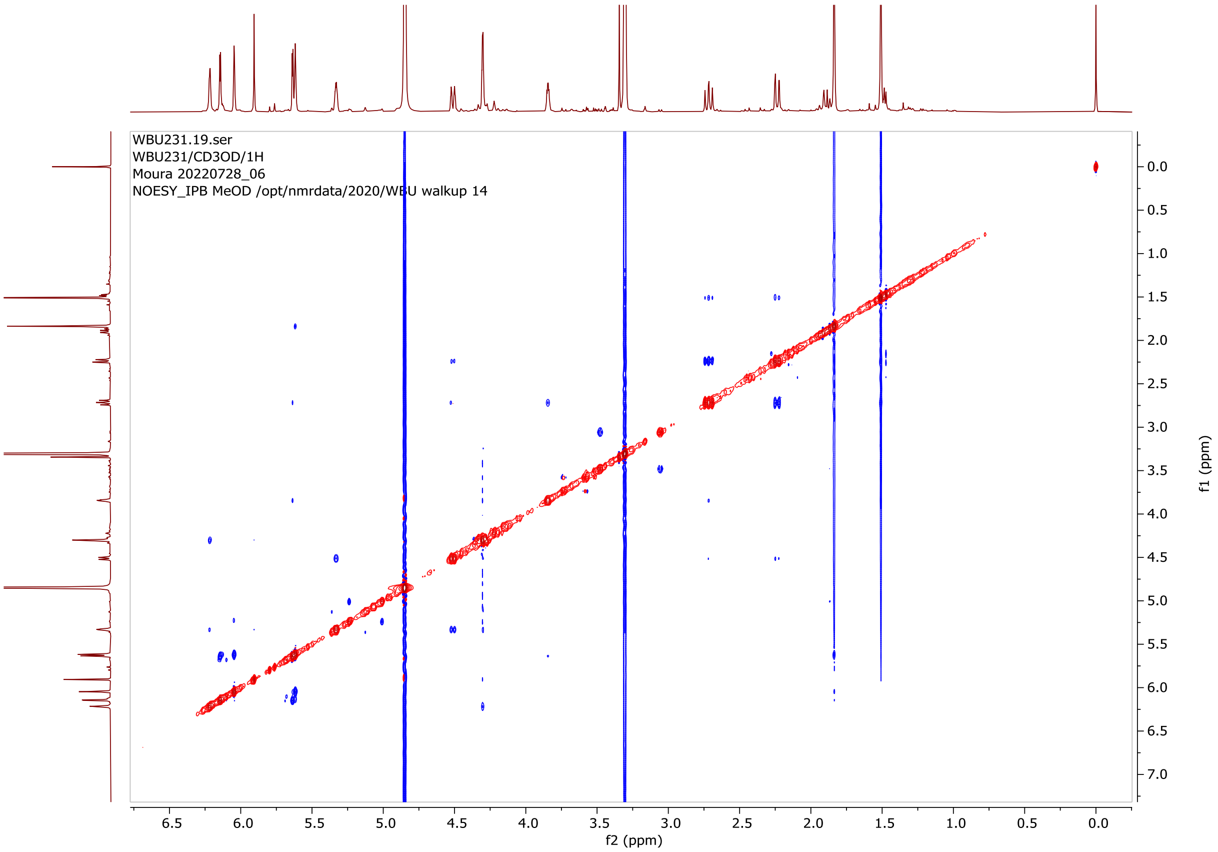

Figure S27. (^{1}H-^{1}H) NOESY NMR spectra (δ_H -1.0 to 7.5 ppm; δ_H -0.5 to 7.0 ppm) of goyazensolide.
